# Synthetic approach to 2-alkyl-4-quinolones and 2-alkyl-4-quinolone-3-carboxamides based on common β-keto amide precursors

**DOI:** 10.3762/bjoc.19.132

**Published:** 2023-11-23

**Authors:** Yordanka Mollova-Sapundzhieva, Plamen Angelov, Danail Georgiev, Pavel Yanev

**Affiliations:** 1 Department of Organic Chemistry, University of Plovdiv Paisii Hilendarski, 24 Tsar Asen Str., 4000 Plovdiv, Bulgaria; 2 Department of Biochemistry and Microbiology, University of Plovdiv Paisii Hilendarski, 24 Tsar Asen Str., 4000 Plovdiv, Bulgaria

**Keywords:** antibacterial, β-keto amides, enaminones, 4-quinolones, quorum sensing

## Abstract

β-Keto amides were used as convenient precursors to both 2-alkyl-4-quinolones and 2-alkyl-4-quinolone-3-carboxamides. The utility of this approach is demonstrated with the synthesis of fourteen novel and four known quinolone derivatives, including natural products of microbial origin such as HHQ and its C_5_-congener. Two compounds with high activity against *S. aureus* have been identified among the newly obtained quinolones, with MICs ≤ 3.12 and ≤ 6.25 µg/mL, respectively.

## Introduction

Among the vast number of biologically active quinoline derivatives [1–2], the subclass of 4-quinolones (also referred to as 4-oxo-1,4-dihydroquinolines, quinolin-4(1*H*)-ones, or 4-hydroxyquinolines) is of great importance with its rich variety of bioactive compounds. Perhaps the most prominent examples in this regard are the fluoroquinolone antimicrobials [3] – a remarkably successful drug class, used to treat bacterial infections caused by both Gram-positive and Gram-negative bacteria [4]. Other notable 4-quinolones of synthetic origin are ivacaftor [5] and elvitegravir [6], drugs used to treat cystic fibrosis and HIV infection, respectively.

A plethora of 4-quinolones with various substitution patterns and biological activities have been isolated from natural sources. This includes plant-derived alkaloids such as graveoline [7], evocarpine [8], leiokinine [9], evollionine C [10], leptomerine [11], and punarivine [12]. The fruit of *Evodia rutaecarpa* is a particularly rich source of 4-quinolones with long-chain substituents at position 2. Various alkaloids isolated from this source have been shown to possess anti-*Helicobacter pylori* activity [13], inhibitory effects on monoamine oxidase [14], cytotoxicity against cancer cell lines [15], activity against nuclear factor of activated T cells [16], and anti-inflammatory activity [17]. Alkaloids with similar structure and anti-inflammatory activity have been isolated from another member of the Rutaceae family – *Zanthoxylum avicennae* [18]. Inhibition of hepatitis C virus replication by 2-nonyl-4-quinolone, isolated from *Ruta angustifolia* leaves, has also been reported [19].

Another significant group of natural 4-quinolones are those of microbial origin. The function of these compounds in the microbial world is a matter of great research interest, with many reviews published in the recent years [20–22]. Some of the compounds are known to act as antibiotics [23–26], while others function as quorum-sensing signal molecules which regulate the production and release of virulence factors in bacteria, thus helping them to colonize niches and gain advantage over competitors [27–28]. Interference with this complicated communication mechanism is considered a viable strategy of combating bacterial infections and consequently a lot of research efforts have been devoted to it [29–33]. Many of the 4-quinolones produced by the Gram-negative opportunistic pathogen *Pseudomonas aeruginosa* and related species feature a saturated long-chain substituent at position 2 and are sometimes referred to as pseudanes [34–35]. *Pseudomonas aeruginosa* alone produces over 50 different quinolones, among which the most extensively studied is 2-heptyl-4-quinolone (HHQ) and its oxygenated derivatives 2-heptyl-3-hydroxy-4-quionolone (PQS) and 4-hydroxy-2-heptylquinoline-*N*-oxide (HQNO) [27,36–38].

Considering the importance of 4-quinolones as potential drugs and biological probes, it is not surprising that the development of methods for their synthesis is a very active area of research. Recent contributions to the synthesis of 4-quinolones made use of phosphine-mediated redox cyclization of 1-(2-nitroaryl)prop-2-ynones [39], palladium-catalyzed carbonylative cyclization of 2-bromonitrobenzenes and alkynes [40], TsCl-mediated domino reaction of chromone-3-carboxaldehydes and amines [41], Pd-catalyzed redox-neutral C–N coupling reaction of iminoquinones with electron-deficient alkenes [42], NH_3_ insertion into *o*‑haloarylynones [43], gold(III)-catalyzed azide-yne cyclization [44], Michael/Truce-Smiles rearrangement cascade [45], and base-promoted annulations with isatoic anhydrides [46]. Many other contributions in this field up to 2019 have been extensively reviewed [47–49], with special attention to the total synthesis and functional analysis of 2-alkyl-4-quinolones as microbial signaling molecules [50–51].

Despite the variety of synthetic approaches to the construction and functionalization of the 4-quinolone ring system, most of the recent studies related to microbial 2-alkyl-4-quinolones relied on variations of the age-old Conrad–Limpach and Camps methods for the construction of the heterocyclic quinolone core [26,36,52–55]. These methods usually give poor overall yields of the target quinolone products and require rather harsh conditions during the ring-forming step, such as prolonged heating in Ph_2_O (270 °C) or in dioxane/NaOH (110 °C), respectively. This, along with the importance of the C-3 substitution in analogues of microbial behavioral modulators [54,56], prompted us to investigate a new synthetic approach that could provide a straightforward access to both 2-alkyl-4-quinolones and 2-alkyl-4-quinolone-3-carboxamides. Our approach falls within the broader methodological group of reductive cyclizations of *o*-nitrobenzoyl ketones [57–58], enamines [59–60], or isoxazoles [61]. The scope of these reductive cyclizations is limited by the availability of the necessary intermediates and has remained largely underexplored, especially with regard to 4-quinolones with long-chain substituents at the C-2 position. As a way of expanding the scope of this methodology, we resorted to the α-*C*-acylation of β-enamino amides, a reliable reaction, the utility of which we have already demonstrated in other contexts [62–63].

## Results and Discussion

**Scheme 1:**
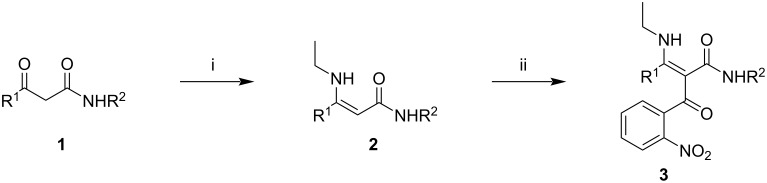
Preparation of α-(*o*-nitrobenzoyl)-β-enamino amides **3**. Reagents and conditions: i) EtNH_2_ (70% aq, 1.05–1.15 equiv), CH_2_Cl_2_, Na_2_SO_4_, 24 h, rt; ii) NMM (1 equiv), DMAP (0.2 equiv), *o*-nitrobenzoyl chloride (1 equiv), CH_2_Cl_2_, 2 h, rt.

As the starting point of our synthetic experiments we used a set of β-keto amides **1**. One of these compounds (**1g**) was acquired from a commercial supplier, others (**1h** and **1i**) were prepared by acetoacetylation of the corresponding amine [64], and the remaining ones (**1a**–**f**) were prepared according to our previously published method [65–66]. The intermediate β-enamino amides **2** are easily available by condensation of the corresponding β-keto amide **1** and an amine (Scheme 1, conditions i). As the amine here plays only an auxiliary role, for the purpose of this research we opted for inexpensive ethylamine. Compounds **2** were obtained by simply stirring a dichloromethane solution of the corresponding keto amide **1** with a slight excess of 70% aqueous ethylamine over Na_2_SO_4_ and were used directly in the next step, without purification. These compounds are highly reactive at their α-position towards acylating reagents and this provides an opportunity to prepare the key *o*-nitrobenzoyl intermediates **3** in a reaction with *o*-nitrobenzoyl chloride (Scheme 1, conditions ii). The acylation of **2** to **3** proceeded with variable yields, depending on the substituents R^1^ and R^2^. Derivatives **2** with a primary carboxamide group (R^2^ = H) gave generally lower and poorly reproducible yields of the desired products **3**. On the other hand, when R^2^ was aryl or benzyl the yields of **3** over two steps were very good, in the range of 75–92% (Table 1). The R^1^ substituent influenced the yield of **3** to a lower extent, but with an unfavorable effect of sterically bulkier substituents. Any α-substitution in R^1^ drove the yields of **3** below 50% and for this reason isolation and further elaboration of such products were considered impractical.

**Table 1:** Yields of α-(*o*-nitrobenzoyl)-β-enamino amides **3** prepared according to Scheme 1.

**3**	R^1^	R^2^	Yield **3** [%]^a^

**a**	*n-*C_3_H_7_	C_6_H_5_	90
**b**	iBu	C_6_H_5_	75
**c**	*n-*C_5_H_11_	C_6_H_5_	89
**d**	*n-*C_7_H_15_	C_6_H_5_	88
**e**	*n-*C_7_H_15_	*p-*C_6_H_4_OCH_3_	86
**f**	*n-*C_7_H_15_	*p-*C_6_H_4_Cl	91
**g**	CH_3_	*p-*C_6_H_4_Cl	90
**h**	CH_3_	*p-*C_6_H_4_OCH_3_	92
**i**	CH_3_	CH_2_C_6_H_5_	90

^a^Over two steps, without purification of intermediate **2**.

**Scheme 2:**
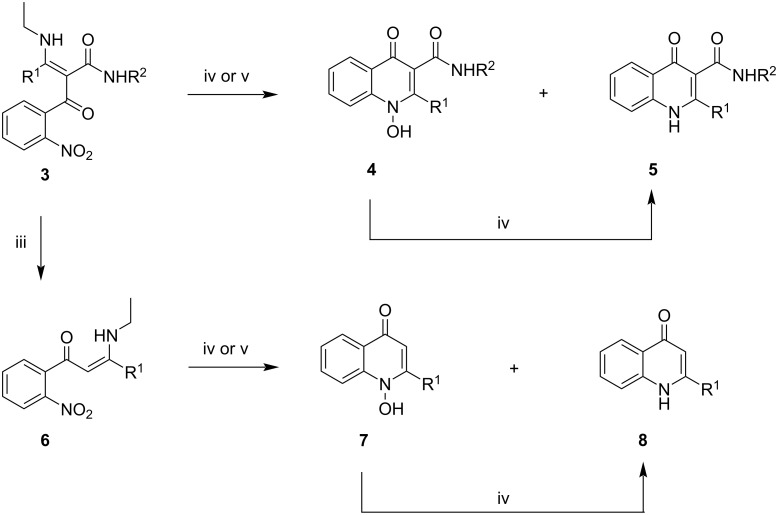
Alternative manipulations of intermediates **3**, leading to either 2-alkyl-4-quinolones **8** (via enaminoketones **6**) or 2-alkyl-4-quinolone-3-carboxamides **5** (by direct reduction/cyclocondensation). Reagents and conditions: iii) H_3_PO_4_, 60 °C, 90 min; iv) Zn/AcOH/CH_2_Cl_2_, rt, overnight; v) HCOONH_4_, Pd/C, CH_3_OH, rt. See main text for details.

Once prepared, the key intermediates **3** could be transformed either directly to 2-alkyl-4-quinolone-3-carboxamides **5** or to 2-alkyl-4-quinolones **8**, after an additional decarbamoylative step (Scheme 2). The decarbamoylation of compounds **3a**–**d** was carried out by heating at 60 °C in neat H_3_PO_4_ for 90 minutes [62] and gave the corresponding β-enaminoketones **6a**–**d** in good yields (Table 2). The NMR spectra of compounds **6** in DMSO-*d*_6_ in all cases indicated a mixture of *Z*/*E* isomers in approximately 85:15 ratio. The same spectra in CDCl_3_ showed broad coalescent signals for the characteristic vinyl CH protons, which is indicative of a dynamic equilibrium between the isomers.

**Table 2:** Yields of β-enaminoketones **6** prepared by decarbamoylation of intermediates **3**, according to Scheme 2:

**6**	R^1^	Yield **6** [%]

**a**	*n-*C_3_H_7_	90
**b**	iBu	91
**c**	*n-*C_5_H_11_	91
**d**	*n-*C_7_H_15_	93

For both types of nitro intermediates **3** and **6** the final ring-forming step required reduction of the nitro group with subsequent cyclization of the reduced intermediate (Scheme 2, conditions iv). We tried to carry out these reactions either with Zn in acetic acid/dichloromethane or by transfer hydrogenation with ammonium formate in the presence of Pd on charcoal. Both types of reductive conditions presented a challenge with regard to the chemoselectivity of the desired transformation, as they initially gave mixtures of 4-quinolones **5** or **8**, respectively, and their corresponding *N*-hydroxy derivatives **4** or **7**, respectively. Such a result is not surprising, considering that the reduction of the aromatic nitro derivatives **3** and **6** proceeds through the corresponding hydroxylamines, capable of intramolecular cyclization to products **4** or **7**. Fortunately, under Zn/AcOH reductive conditions this was resolved by extending the duration of the reaction to 18–24 h, providing enough time for compounds **4**/**7** to get reduced to quinolones **5**/**8**, which were isolated in good yields (Table 3 and Table 4).

**Table 3:** Yields of 2-alkyl-4-quinolone-3-carboxamides **5**, prepared according to Scheme 2.

**5**	R^1^	R^2^	Yield **5** [%]

**a**	*n-*C_3_H_7_	C_6_H_5_	90
**b**	iBu	C_6_H_5_	56
**c**	*n-*C_5_H_11_	C_6_H_5_	63
**d**	*n-*C_7_H_15_	C_6_H_5_	90
**e**	*n-*C_7_H_15_	*p-*C_6_H_4_OCH_3_	72
**f**	*n-*C_7_H_15_	*p-*C_6_H_4_Cl	83
**g**	CH_3_	*p-*C_6_H_4_Cl	92
**h**	CH_3_	*p-*C_6_H_4_OCH_3_	92
**i**	CH_3_	CH_2_C_6_H_5_	79

**Table 4:** Yields of 2-alkyl-4-quinolones **8**, prepared according to Scheme 2:

**8**	R^1^	Yield **8** [%]

**a**	*n-*C_3_H_7_	72
**b**	iBu	74
**c**	*n-*C_5_H_11_	90
**d**	*n-*C_7_H_15_	90

In the case of the Pd-catalyzed transfer hydrogenation of intermediates **3** the yields of products **5** in most cases were lower than those obtained with Zn/AcOH, regardless of the reaction duration. On the other hand, limiting the reaction time to 60–90 min under these conditions allowed some of the *N*-hydroxy derivatives **4** to be isolated in good yield (Table 5), even though it did not entirely prevent the formation of products **5**. Palladium catalysis was not appropriate for the hydrogenation of compounds **3f** and **3g**, because of concomitant reduction at the C–Cl bond.

**Table 5:** Yields of 1-hydroxy-2-alkyl-4-quinolone-3-carboxamides **4**, prepared according to Scheme 2.

**4**	R^1^	R^2^	Yield **4** [%]

**a**	*n-*C_3_H_7_	C_6_H_5_	57
**b**	iBu	C_6_H_5_	75
**c**	*n-*C_5_H_11_	C_6_H_5_	60
**d**	*n-*C_7_H_15_	C_6_H_5_	70
**e**	*n-*C_7_H_15_	*p-*C_6_H_4_OCH_3_	64

Intermediates **6**, similarly to compounds **3**, gave mixtures of products **7**/**8** under palladium-catalyzed transfer hydrogenation conditions. In contrast to **3**, however, limiting the reaction time here did not help to develop a preparatively useful procedure for a preferential isolation of *N*-hydroxy derivatives **7**. Further experiments for palladium-catalyzed hydrogenation with H_2_ at atmospheric pressure did not show any advantage over the transfer hydrogenation conditions.

Overall, the described synthetic approach (Scheme 1 and Scheme 2) allowed us to prepare in an operationally simple manner 2-alkyl-4-quinolones **8a**–**d**, all of which are known from the literature [25,36,61,67–68] and two of them are natural products of microbial origin (**8c** [69] and **8d** [70]). More importantly, the utility of the approach was demonstrated with the synthesis of the novel 2-alkyl-4-quinolone-3-carboxamides **5a**–**i** and some of their *N*-hydroxy derivatives **4a**–**e**. Compounds of this type with C-2 substitution other than methyl [71] have not been previously described.

All of the obtained products were screened for antimicrobial activity at a concentration of 1 mg/mL against *S. aureus* and *E. coli*, using the hole-plate method in Mueller–Hinton agar, with 100 µg loading of each compound in 100 µL DMSO (Table 6). Interestingly, at this concentration most of the compounds showed weak to moderate activity against *E. coli*, while *S. aureus* was inhibited only by C_5_ and C_7_-substituted analogs. Among the novel compounds, only compounds **4d** and **4e** gave inhibition zones of more than 20 mm and were further analyzed to determine their minimum inhibitory concentrations (MIC) by serial broth dilutions [72]. The MICs measured for **4d** and **4e** were ≤6.25 µg/mL and ≤3.12 µg/mL, respectively, with a MIC ≤ 0.78 µg/mL for levofloxacin as the positive control.

**Table 6:** Antibacterial activity of the synthesized quinolone derivatives **4**, **5**, and **8**.

Compound^a^	Sterile zone diameter (mm)^b^	

	*S. aureus* ATCC 25923	*E. coli* ATCC 25922

**4b**	–	17
**4d**	27	16
**4e**	22	–
**5a**	–	16
**5b**	–	15
**5c**	–	14
**5d**	19	14
**5e**	15	16
**5f**	15	15
**8a**	–	16
**8b**	–	15
**8c**	18	15
**8d**	21	13

^a^Compounds giving sterile zones of less than 10 mm are not listed. ^b^Including well diameter of 8 mm.

## Conclusion

In conclusion, we have demonstrated that β-keto amides and 2-nitrobenzoyl chloride can be used as convenient precursors to a variety of 4-quinolone derivatives. The described approach is realized in a small number of steps, under mild conditions, and allows easy installation of long-chain substituents at the C-2 position of the quinolone core. These characteristics of the synthetic method could be particularly attractive in the search of novel mimics of the *Pseudomonas* quorum-sensing signal molecules. The high activity of compounds **4d** and **4e** against *S. aureus* provides a good lead for further structural optimizations.

## Supporting Information

File 1 Full experimental details and analytical data.

File 2 Processed NMR spectra.
