# Supplementary material for: Synthetic approach to 2-alkyl-4-quinolones and 2-alkyl-4-quinolone-3-carboxamides based on common β-keto amide precursors
Source: Beilstein J Org Chem. 2023 Nov 23;19:1804–10. doi: 10.3762/bjoc.19.132 (PMC10682542; doi:10.3762/bjoc.19.132)
Supplement: File 1 — Full experimental details and analytical data. [file Beilstein_J_Org_Chem-19-1804-s001.pdf]

BEILSTEIN JOURNAL OF ORGANIC CHEMISTRY

Supporting Information

for

Synthetic approach to 2-alkyl-4-quinolones and 2-alkyl-4-quinolone-3-carboxamides based on common β-keto amide precursors

Yordanka Mollova-Sapundzhieva, Plamen Angelov, Danail Georgiev and Pavel Yanev

Beilstein J. Org. Chem. **2023,** *19,* 1804–1810. doi:10.3762/bjoc.19.132

Full experimental details and analytical data

License and Terms: This is a supporting information file under the terms of the Creative Commons Attribution License (https://creativecommons.org/licenses/by/4.0). Please note that the reuse, redistribution and reproduction in particular requires that the author(s) and source are credited and that individual graphics may be subject to special legal provisions.
The license is subject to the *Beilstein Journal of Organic Chemistry* terms and conditions: (https://www.beilstein-journals.org/bjoc/terms)

General

Unless otherwise noted, reagents and solvents were purchased from Sigma-Aldrich, Darmstadt, Germany, and were used as supplied. NMR spectra were run on Bruker NEO 400 (400/100 MHz $^{1}H/^{13}C$) or Bruker Avance AV600 (600/150 MHz $^{1}H/^{13}C$) spectrometers at BAS-IOCCP, Sofia. Chemical shifts (δ, ppm) are reported downfield from TMS. High resolution mass spectral measurements were performed on a Waters Synapt XS mass spectrometer. TLC was done on aluminium-backed silica gel 60 sheets (Merck) with $KMnO_4$ staining; Melting point measurements were done in capillary tubes on a KRÜSS M5000 automatic mp meter and are not corrected.

Synthesis of β-enamino amides (2), general procedure:

Ethylamine (70% aq. solution, 0.45 mL, 5.7 mmol) was added to a solution of the corresponding β-keto amide **1** (5 mmol) in CH_2Cl_2 (15–20 mL). The mixture was magnetically stirred in a tightly closed vial for 4 h at rt, then, anhydrous sodium sulfate was added and the stirring was continued overnight at rt. Then, the sulfate was filtered off and the solvent was evaporated under reduced pressure. To remove any residual solvent or ethylamine, the crude oily residue was kept under vacuum (10 millibars) for 30 min at 40 °C and was then used directly in the next step, without any further purification. Compounds **2** are prone to hydrolysis and cannot be chromatographed on silica gel.

Synthesis of α-(*o*-nitrobenzoyl)-β-enamino amides (3), general procedure:

2-Nitrobenzoyl chloride (0.13 mL, 1 mmol) was slowly added to a magnetically stirred solution containing the corresponding crude enamino amide **2** (1 mmol), 4-methylmorpholine (0.11 mL, 1 mmol) and DMAP (24 mg, 0.2 mmol) in CH_2Cl_2 (20 mL). The mixture was left to stir for 2 h at rt, then, it was transferred to a separatory funnel with additional 30 mL of CH_2Cl_2 and washed with dilute aqueous (20:1) HCl. The aqueous layer was extracted again with 25 mL of CH_2Cl_2, the combined organic layers were dried with anhydrous sodium sulfate, the drying agent was removed by filtration, and the solvent was distilled off. The residue was purified by column chromatography on silica gel, using Et_2O as the eluent.

*3-Ethylamino-2-(2-nitrobenzoyl)-hex-2-enoic acid phenylamide (**3a**):* Yield 0.343 g (90%), m.p. 104 °C; ^{1}H-NMR (400 MHz, DMSO-d_6, δ ppm, *J* Hz): 0.89 (t, *J* = 7.3, 3H), 1.27 (t, *J* = 7.2, 3H), 1.62 (m, 2H), 2.45 (m, 2H), 3.50 (m, 2H), 6.95 (m, 1H), 7.16 (m, 2H), 7.25 (m, 2H), 7.48 (m, 2H), 7.65 (td, *J* = 7.5, *J* = 1.2, 1H), 7.96 (dd, *J* = 8.2, *J* = 0.9, 1H), 9.68 (br s, 1H), 11.64 (t, *J* = 5.4, 1H); ^{13}C-NMR (100 MHz, DMSO-d_6, δ ppm): 14.6, 15.8, 21.7, 31.8, 38.1, 107.6, 119.7, 123.7, 124.3, 128.8, 128.9, 129.8, 133.9, 137.7, 139.4, 146.4, 167.2, 169.2, 186.1; HRMS *m/z* (ES+): calcd. for $C_{21}H_{23}N_3NaO_4^+$ $[M+Na]^+$ 404.1581, found 404.1589

*3-Ethylamino-5-methyl-2-(2-nitrobenzoyl)-hex-2-enoic acid phenylamide (**3b**):* Yield 0.296 g (75%), oil; ^{1}H-NMR (600 MHz, DMSO-d_6, δ ppm, *J* Hz): 0.92 (d, *J* = 6.6, 6H), 1.29 (t, *J* = 7.1, 3H), 1.97 (m, 1H), 2.50 (d, *J* = 7.3, 2H), 3.54 (m, 2H), 6.56 (m, 1H), 7.18 (m, 2H), 7.27 (m, 2H), 7.49 (m, 2H), 7.67 (m, 1H), 7.99 (m, 1H), 9.70 (br s, 1H), 11.90 (t, *J* = 5.4, 1H); ^{13}C-NMR (150 MHz, DMSO-d_6, δ ppm): 15.8, 22.8, 27.9, 37.8, 38.5, 107.9, 119.6, 123.6, 124.2, 128.6, 128.9, 129.7, 134.0, 137.9, 139.6, 146.4, 167.2, 168.9, 186.3; HRMS *m/z* (ES+): calcd. for $C_{22}H_{25}N_3NaO_4^+$ $[M+Na]^+$ 418.1737, found: 418.1742

*3-Ethylamino-2-(2-nitrobenzoyl)-oct-2-enoic acid phenylamide (**3c**):* Yield 0.364 g (89%), oil; ^{1}H-NMR (400 MHz, DMSO-d_6, δ ppm, *J* Hz): 0.79 (t, *J* = 7.1, 3H), 1.23 (m, 4H), 1.27 (t, *J* = 7.2, 3H), 1.61 (m, 2H), 2.45 (m, 2H), 3.50 (m, 2H), 6.94 (m, 1H), 7.16 (m, 2H), 7.25 (m, 2H), 7.50 (m, 2H), 7.66 (td, *J* = 7.5, *J* = 1.2, 1H), 7.96 (dd, *J* = 8.2, *J* = 0.9, 1H), 9.69 (br s, 1H), 11.62 (t, *J* = 5.5, 1H); ^{13}C-NMR (100 MHz, DMSO-d_6, δ ppm): 14.1, 15.8, 22.0, 27.7, 29.8, 31.7,

38.1, 107.6, 119.7, 123.7, 124.3, 128.8, 128.9, 129.8, 133.9, 137.7, 139.5, 146.5, 167.1, 169.4, 186.1; HRMS *m/z* (ES+): calcd. for $C_{23}H_{27}N_3NaO_4^+$ $[M+Na]^+$ 432.1894, found 432.1880

*3-Ethylamino-2-(2-nitrobenzoyl)-dec-2-enoic acid phenylamide (**3d**):* Yield 0.385 g (88%) oil; ^{1}H-NMR (400 MHz, DMSO-d_6, δ ppm, *J* Hz): 0.78 (t, *J* = 7.0, 3H), 1.16 (m, 8H), 1.27 (t, *J* = 7.2, 3H), 1.61 (m, 2H), 2.45 (m, 2H), 3.50 (m, 2H), 6.94 (m, 1H), 7.16 (m, 2H), 7.26 (m, 2H), 7.50 (m, 2H), 7.66 (td, *J* = 7.5, *J* = 1.1, 1H), 7.96 (dd, *J* = 8.2, *J* = 0.9, 1H), 9.70 (br s, 1H), 11.62 (t, *J* = 5.5, 1H); ^{13}C-NMR (100 MHz, DMSO-d_6, δ ppm): 14.3, 15.7, 22.4, 28.0, 28.5, 29.5, 29.8, 31.4, 38.0, 107.6, 119.6, 123.6, 124.3, 128.8, 128.9, 129.8, 133.9, 137.7, 139.5, 146.5, 167.1, 169.0, 186.0; HRMS *m/z* (ES+): calcd. for $C_{25}H_{31}N_3NaO_4^+$ $[M+Na]^+$ 460.2207, found 460.2217

*3-Ethylamino-2-(2-nitrobenzoyl)-dec-2-enoic acid (4-methoxyphenyl)-amide (**3e**):* Yield 0.402 g (86%), oil; ^{1}H-NMR (400 MHz, DMSO-d_6, δ ppm, *J* Hz): 0.78 (t, *J* = 7.0, 3H), 1.16 (m, 8H), 1.26 (t, *J* = 7.2, 3H), 1.60 (m, 2H), 2.44 (m, 2H), 3.48 (m, 2H), 3.65 (s, 3H), 6.73 (m, 2H), 7.14 (m, 2H), 7.49 (m, 2H), 7.66 (td, *J* = 7.5, *J* = 1.0, 1H), 7.96 (m, 1H), 9.52 (br s, 1H), 11.59 (t, *J* = 5.4, 1H); ^{13}C-NMR (100 MHz, DMSO-d_6, δ ppm): 14.3, 15.8, 22.4, 28.0, 28.5, 29.5, 29.7, 31.4, 38.0, 55.6, 107.6, 114.0, 121.3, 124.3, 128.8, 129.8, 132.6, 133.9, 137.7, 146.5, 155.7, 166.7, 169.2, 185.9; HRMS *m/z* (ES+): calcd. for $C_{26}H_{33}N_3NaO_5^+$ $[M+Na]^+$ 490.2312, found 490.2326

*3-Ethylamino-2-(2-nitrobenzoyl)-dec-2-enoic acid (4-chlorophenyl)-amide (**3f**):* Yield 0.430 g (91%), oil; ^{1}H-NMR (400 MHz, DMSO-d_6, δ ppm, *J* Hz): 0.77 (t, *J* = 7.0, 3H), 1.14 (m, 8H), 1.27 (t, *J* = 7.2, 3H), 1.59 (m, 2H), 2.44 (m, 2H), 3.50 (m, 2H), 7.22 (m, 2H), 7.32 (m, 2H), 7.49 (m, 2H), 7.66 (m, 1H), 7.96 (m, 1H), 9.87 (br s, 1H), 11.62 (t, *J* = 5.6, 1H); ^{13}C-NMR (100 MHz, DMSO-d_6, δ ppm): 14.3, 15.8, 22.4, 27.9, 28.4, 29.4, 29.7, 31.4, 38.1, 107.4, 120.9, 124.3, 127.2, 128.8, 129.9, 133.9, 137.6, 138.5, 146.4, 167.3, 169.4, 186.1; HRMS *m/z* (ES+): calcd. for $C_{25}H_{30}ClN_3NaO_4^+$ $[M+Na]^+$ 494.1817, found 494.1825

*3-Ethylamino-2-(2-nitrobenzoyl)-but-2-enoic acid (4-chlorophenyl)-amide (**3g**):* Yield 0.350 g (90%), m.p. 167 °C; ^{1}H-NMR (400 MHz, DMSO-d_6, δ ppm, *J* Hz): 1.26 (t, *J* = 7.2, 3H), 2.14 (s, 3H), 3.48 (m, 2H), 7.24 (m, 2H), 7.37 (m, 2H), 7.45 (dd, *J* = 7.5, *J* = 1.2, 1H), 7.51 (m, 1H), 7.66 (td, *J* = 7.5, *J* = 1.2, 1H), 7.98 (dd, *J* = 8.2, *J* = 0.9, 1H), 9.94 (br s, 1H), 11.66 (br s, 1H); ^{13}C-NMR (100 MHz, DMSO-d_6, δ ppm): 15.3, 17.1, 38.3, 107.8, 121.0, 124.3, 127.2, 128.6, 128.9, 129.9, 134.0, 137.8, 138.5, 146.5, 167.3, 169.0, 186.3; HRMS *m/z* (ES+): calcd. for $C_{19}H_{18}ClN_3NaO_4^+$ $[M+Na]^+$ 410.0878, found 410.0884

*3-Ethylamino-2-(2-nitrobenzoyl)-but-2-enoic acid (4-methoxyphenyl)-amide (**3h**):* Yield 0.353 g (92%), m.p. 125 °C; ^{1}H-NMR (400 MHz, DMSO-d_6, δ ppm, *J* Hz): 1.26 (t, *J* = 7.2, 3H), 2.14 (s, 3H), 3.48 (m, 2H), 3.67 (s, 3H), 6.76 (d, *J* = 8.5, 2H), 7.2 (d, *J* = 8.5, 2H), 7.47 (dd, *J* = 7.5, *J* = 1.2, 1H), 7.51 (m, 1H), 7.67 (td, *J* = 7.5, *J* = 1.2, 1H), 7.98 (dd, *J* = 8.2, *J* = 0.8, 1H), 9.61 (br s, 1H), 11.63 (br s, 1H); ^{13}C-NMR (100 MHz, DMSO-d_6, δ ppm): 15.3, 17.1, 38.3, 55.6, 106.5, 114.1, 121.3, 124.3, 128.7, 129.8, 132.7, 133.9, 137.9, 146.5, 155.7, 166.8; HRMS *m/z* (ES+): calcd. for $C_{20}H_{21}N_3NaO_5^+$ $[M+Na]^+$ 406.1373, found 406.1379

*3-Ethylamino-2-(2-nitrobenzoyl)-but-2-enoic acid benzylamide (**3i**):* Yield 0.330 g (90%), m.p. 126 °C; ^{1}H-NMR (400 MHz, DMSO-d_6, δ ppm, *J* Hz): 1.22 (t, *J* = 7.2, 3H), 2.07 (s, 3H), 3.43 (m, 2H), 4.07 (br s, 2H), 6.84 (br s, 2H), 7.16 (m, 3H), 7.37 (dd, *J* = 7.4, *J* = 1.4, 1H), 7.60 (m, 2H), 7.98 (dd, *J* = 8.2, *J* = 1.1, 1H), 8.04 (br s, 1H), 11.63 (br s, 1H); ^{13}C-NMR (100 MHz, DMSO-d_6, δ ppm): 15.2, 17.0, 38.2, 43.0, 107.3, 124.3, 127.0, 127.5, 128.5, 128.7, 129.8, 133.9, 136.4, 139.6, 146.7, 165.9, 168.6, 185.7; HRMS *m/z* (ES+): calcd. for $C_{20}H_{21}N_3NaO_4^+$ $[M+Na]^+$ 390.1424, found 390.1430

Synthesis of β-enaminoketones (6), general procedure:

To 300 mg of the corresponding intermediate **3** in a glass vial was added H_3PO_4 (4–5 g). The mixture was heated to 60 °C and kept at this temperature while being magnetically stirred in the course of 90 min. The size of the magnetic stirring bar is advisable to correspond as close as possible to the diameter of the glass vial, because this greatly facilitates the initial homogenization of the viscous reaction mixture. Intense foaming and gas

evolution is to be expected in the beginning of the reaction. After completion of the reaction (90 min), the vial was cooled to rt with tap water and the contents were rinsed and poured into a separatory funnel containing 50–70 mL of water. The product was extracted in CH_2Cl_2 (2 × 40 mL), the combined organic layers were dried (Na_2SO_4) and the solvent was removed under reduced pressure. The crude products were purified by column chromatography on silica gel with Et_2O/petrol 1:1 as the eluent. All products were obtained as viscous yellowish oils. ^{1}H NMR spectra of products **6** in DMSO-d_6 indicated *Z*/*E* isomeric mixtures, with approximate ratio of 85:15 in all cases. The major form was determined to be *Z* on the basis of NOESY data for compound **6b**. Only NMR signals corresponding to the major *Z* isomers are listed below.

*3-Ethylamino-1-(2-nitrophenyl)-hex-2-en-1-one (**6a**):* Yield 0.186 g (90%), oil; ^{1}H-NMR (400 MHz, $CDCl_3$, δ ppm, *J* Hz): 1.03 (t, *J* = 7.4, 3H), 1.32 (t, *J* = 7.3, 3H), 1.64 (m, 2H), 2.29 (m, 2H), 3.40 (m, 2H), 5.22 (s, 1H), 7.47 (m, 1H), 7.56 (m, 2H), 7.81 (m, 1H), 11.09 (br s, 1H); ^{13}C-NMR (100 MHz, $CDCl_3$, δ ppm, *J* Hz): 13.9, 15.3, 21.2, 34.1, 37.8, 92.9, 123.8, 128.9, 129.3, 132.4, 138.0, 148.0, 169.6, 186.2; ^{1}H-NMR (400 MHz, DMSO-d_6, δ ppm, *J* Hz): 0.97 (t, *J* = 7.4, 3H), 1.20 (t, *J* = 7.2, 3H), 1.57 (m, 2H), 2.34 (m, 2H), 3.40 (m, 2H), 5.36 (s, 1H), 7.55 – 7.84 (m, 5H), 10.95 (t, *J* = 5.2, 1H); ^{13}C-NMR (100 MHz, DMSO-d_6, δ ppm, *J* Hz): 14.2, 15.7, 21.1, 33.6, 37.6, 92.3, 124.1, 129.1, 130.7, 132.8, 136.9, 148.9, 170.2, 184.6; HRMS *m/z* (ES+): calcd. for $C_{14}H_{18}N_2NaO_3^+$ [M+Na]$^+$ 285.1210, found 285.1218

*3-Ethylamino-5-methyl-1-(2-nitrophenyl)-hex-2-en-1-one (**6b**):* Yield 0.191 g (91%), oil; ^{1}H-NMR (400 MHz, DMSO-d_6, δ ppm, *J* Hz): 0.97 (d, *J* = 6.6, 6H), 1.19 (t, *J* = 7.2, 3H), 1.91 (m, 1H), 2.24 (d, *J* = 7.3, 2H), 3.40 (m, 2H), 5.31 (s, 1H), 7.64 (m, 3H), 7.82 (m, 1H), 11.00 (t, *J* = 5.4, 1H); ^{13}C-NMR (100 MHz, DMSO-d_6, δ ppm): 15.8, 22.7, 27.5, 37.8, 40.6, 93.6, 124.1, 129.1, 130.6, 132.9, 137.0, 148.9, 169.3, 184.4; HRMS *m/z* (ES+): calcd. for $C_{15}H_{20}N_2NaO_3^+$ [M+Na]$^+$ 299.1366, found 299.1361

*3-Ethylamino-1-(2-nitrophenyl)-oct-2-en-1-one (**6c**):* Yield 0.194 g (91%), oil; ^{1}H-NMR (400 MHz, DMSO-d_6, δ ppm, *J* Hz): 0.89 (t, *J* = 7.1, 3H), 1.20 (t, *J* = 7.2, 3H), 1.33 (m, 4H), 1.54 (m, 2H), 2.34 (m, 2H), 3.39 (m, 2H), 5.36 (s, 1H), 7.64 (m, 3H), 7.82 (m, 1H), 10.95 (t, *J* = 5.5, 1H); ^{13}C-NMR (100 MHz, DMSO-d_6, δ ppm): 14.3, 15.7, 22.3, 27.5, 31.4, 31.7, 37.6, 92.3, 124.1, 129.1, 130.7, 132.8, 136.9, 148.9, 170.4, 184.7; HRMS *m/z* (ES+): calcd. for $C_{16}H_{22}N_2NaO_3^+$ [M+Na]$^+$ 313.1523, found 313.1523

*3-Ethylamino-1-(2-nitrophenyl)-dec-2-en-1-one (**6d**):* Yield 0.203 g (93%), oil; ^{1}H-NMR (400 MHz, DMSO-d_6, δ ppm, *J* Hz): 0.87 (t, *J* = 7.0, 3H), 1.20 (t, *J* = 7.2, 3H), 1.30 (m, 8H), 1.54 (m, 2H), 2.34 (m, 2H), 3.39 (m, 2H), 5.35 (s, 1H), 7.64 (m, 3H), 7.82 (m, 1H), 10.94 (t, *J* = 5.5, 1H); ^{13}C-NMR (100 MHz, DMSO-d_6, δ ppm): 14.4, 15.7, 22.5, 27.8, 28.8, 29.2, 31.6, 31.8, 37.6, 92.3, 124.1, 129.1, 130.7, 132.8, 136.9, 148.9, 170.4, 184.6; HRMS *m/z* (ES+): calcd. for $C_{18}H_{26}N_2NaO_3^+$ [M+Na]$^+$ 341.1836, found 341.1841

Synthesis of 2-alkyl-4-quinolone-3-carboxamides (5) and 2-alkyl-4-quinolones (8), general procedure:

To the corresponding nitro-intermediate **3** or **6** (1 mmol) dissolved in CH_2Cl_2 (30 mL) was added acetic acid (4 mL) and Zn powder (1 g). The mixture was magnetically stirred overnight (18–24 h) at rt, then, the solids were filtered off with suction and rinsed thoroughly with CH_2Cl_2. The dichloromethane filtrate was transferred to a separatory funnel and was extracted with water (50 mL) and then with saturated aqueous solution of $NaHCO_3$ (25 mL). The organic phase was dried with anhydrous sodium sulfate, the drying agent was filtered off and the solvent was removed under reduced pressure. The crude products were purified by column chromatography on silica gel with Et_2O as the eluent, increasing polarity to Et_2O/MeOH 20:1, where necessary.

4-Oxo-2-propyl-1,4-dihydroquinoline-3-carboxylic acid phenylamide ***(5a)****:* Yield 0.276 g (90%), m.p. 216 ^{o}C; ^{1}H-NMR (400 MHz, DMSO-d_6, δ ppm, *J* Hz): 1.00 (t, *J* = 7.3, 3H), 1.77 (m, 2H), 3.15 (t, *J* = 7.7, 2H), 7.07 (m, 1H), 7.34 (m, 2H), 7.44 (m, 1H), 7.71 (m, 4H), 8.23 (dd, *J* = 8.1, *J* = 1.3, 1H), 12.19 (br s, 1H), 12.25 (s, 1H); ^{13}C-NMR (100 MHz, DMSO-d_6, δ ppm): 14.4, 23.4, 35.5, 112.3, 118.6, 120.1, 123.5, 124.9, 125.2, 125.9, 129.3, 133.1, 138.8, 139.8, 158.7, 164.6, 176.8; HRMS *m/z* (ES+): calcd. for $C_{19}H_{19}N_2O_2^+$ [M+H]$^+$ 307.1441, found 307.1445

2-Isobutyl-4-oxo-1,4-dihydroquinoline-3-carboxylic acid phenylamide ***(5b)****:* Yield 0.180 g (56%), m.p. 252 ^{o}C; ^{1}H-NMR (400 MHz, DMSO-d_6, δ ppm, *J* Hz): 0.95 (d, *J* = 6.7, 6H), 2.17 (m, 1H), 3.11 (d, *J* = 7.3, 2H), 7.06 (m, 1H), 7.34 (m, 2H), 7.44 (m, 1H), 7.72 (m, 4H), 8.24 (dd, *J* = 8.1, *J* = 1.2, 1H), 12.09 (br s, 1H), 12.13 (s, 1H); ^{13}C-NMR (100 MHz, DMSO-d_6, δ ppm): 22.7, 29.2, 41.7, 113.1, 118.7, 120.1, 123.5, 124.9, 125.2, 125.9, 129.3, 133.1, 138.7, 139.8, 157.6, 164.7, 176.6; HRMS *m/z* (ES-): calcd. for $C_{20}H_{19}N_2O_2^-$ [M-H]$^-$ 319.1452, found 319.1450

4-Oxo-2-pentyl-1,4-dihydroquinoline-3-carboxylic acid phenylamide ***(5c)****:* Yield 0.210 g (63%), m.p. 218 ^{o}C; ^{1}H-NMR (400 MHz, DMSO-d_6, δ ppm, *J* Hz): 0.88 (t, *J* = 7.1, 3H), 1.36 (m, 4H), 1.74 (m, 2H), 3.16 (m, 2H), 7.06 (m, 1H), 7.34 (m, 2H), 7.43 (m, 1H), 7.71 (m, 4H), 8.23 (dd, *J* = 8.1, *J* = 1.2, 1H), 12.10 (br s, 1H), 12.23 (s, 1H); ^{13}C-NMR (100 MHz, DMSO-d_6, δ ppm): 14.3, 22.3, 29.6, 31.8, 33.6, 112.3, 118.6, 120.1, 123.5, 124.8, 125.2, 125.9, 129.3, 133.1, 138.9, 139.8, 158.9, 164.6, 176.7; HRMS *m/z* (ES-): calcd. for $C_{21}H_{21}N_2O_2^-$ [M-H]$^-$ 333.1609, found 333.1618

2-Heptyl-4-oxo-1,4-dihydroquinoline-3-carboxylic acid phenylamide ***(5d)****:* Yield 0.326 g (90%), m.p. 201 ^{o}C; ^{1}H-NMR (400 MHz, DMSO-d_6, δ ppm, *J* Hz): 0.83 (t, *J* = 6.8, 3H), 1.27 (m, 6H), 1.39 (m, 2H), 1.73 (m, 2H), 3.15 (m, 2H), 7.06 (m, 1H), 7.33 (m, 2H), 7.43 (m, 1H), 7.70 (m, 4H), 8.23 (dd, *J* = 8.1, *J* = 1.2, 1H), 12.14 (br s, 1H), 12.20 (s, 1H); ^{13}C-NMR (100 MHz, DMSO-d_6, δ ppm): 14.4, 22.5, 28.9, 29.5, 29.9, 31.6, 33.6, 112.4, 118.6, 120.1, 123.5, 124.8, 125.2, 125.9, 129.2, 133.1, 138.9, 139.8, 158.9, 164.6, 176.7; HRMS *m/z* (ES+): calcd. for $C_{23}H_{26}N_2NaO_2^+$ [M+Na]$^+$ 385.1886, found 385.1898

2-Heptyl-4-oxo-1,4-dihydroquinoline-3-carboxylic acid (4-methoxyphenyl)-amide ***(5e)****:* Yield 0.283 g (72%), m.p. 211 ^{o}C; ^{1}H-NMR (400 MHz, DMSO-d_6, δ ppm, *J* Hz): 0.84 (t, *J* = 6.8, 3H), 1.27 (m, 6H), 1.39 (m, 2H), 1.72 (m, 2H), 3.15 (m, 2H), 3.74 (s, 3H), 6.91 (m, 2H), 7.42 (m, 1H), 7.63 (m, 3H), 7.74 (m, 1H), 8.22 (dd, *J* = 8.1, *J* = 1.1, 1H), 12.04 (s, 1H), 12.14 (br s, 1H); ^{13}C-NMR (100 MHz, DMSO-d_6, δ ppm): 14.4, 22.5, 28.9, 29.5, 30.0, 31.6, 33.6, 55.6, 112.5, 114.4, 118.6, 121.5, 124.8, 125.1, 125.9, 132.9, 133.0, 138.9, 155.6, 158.6, 164.2, 176.6; HRMS *m/z* (ES-): calcd. for $C_{24}H_{27}N_2O_3^-$ [M-H]$^-$ 391.2027, found 391.2017

2-Heptyl-4-oxo-1,4-dihydroquinoline-3-carboxylic acid (4-chlorophenyl)-amide ***(5f)****:* Yield 0.330 g (83%), m.p. 183 ^{o}C; ^{1}H-NMR (400 MHz, DMSO-d_6, δ ppm, *J* Hz): 0.83 (t, *J* = 6.8, 3H), 1.27 (m, 6H), 1.38 (m, 2H), 1.72 (m, 2H), 3.13 (m, 2H), 7.37 (m, 2H), 7.43 (m, 1H), 7.65 (d, *J* = 8.1, 1H), 7.74 (m, 3H), 8.21 (dd, *J* = 8.1, *J* = 1.2, 1H), 12.19 (br s, 1H), 12.32 (s, 1H); ^{13}C-NMR (100 MHz, DMSO-d_6, δ ppm): 14.4, 22.5, 28.8, 29.5, 29.9, 31.6, 33.6, 112.2, 118.6, 121.5, 124.9, 125.1, 125.9, 127.0, 129.1, 133.1, 138.7, 138.8, 159.0, 164.7, 176.7; HRMS *m/z* (ES-): calcd. for $C_{23}H_{24}ClN_2O_2^-$ [M-H]$^-$ 395.1532, found 395.1516

2-Methyl-4-oxo-1,4-dihydroquinoline-3-carboxylic acid (4-chlorophenyl)-amide ***(5g)****:* Yield 0.288 g (92%), m.p. 250 ^{o}C, dec.; ^{1}H-NMR (400 MHz, DMSO-d_6, δ ppm, *J* Hz): 2.83 (s, 3H), 7.38 (m, 2H), 7.46 (m, 1H), 7.65 (d, *J* = 8.0, 1H), 7.76 (m, 3H), 8.24 (dd, *J* = 8.1, *J* = 1.2, 1H), 12.36 (br s, 1H), 12.64 (s, 1H); ^{13}C-NMR (100 MHz, DMSO-d_6, δ ppm): 21.7, 111.4, 118.7, 121.6, 125.1, 125.2, 125.9, 126.9, 129.2, 133.3, 138.6, 138.7, 156.3, 164.9, 176.7; HRMS *m/z* (ES+): calcd. for $C_{17}H_{13}ClN_2NaO_2^+$ [M+Na]$^+$ 335.0558, found 335.0563

2-Methyl-4-oxo-1,4-dihydroquinoline-3-carboxylic acid (4-methoxyphenyl)-amide ***(5h)****:* Yield 0.284 g (92%), m.p. 185 ^{o}C; ^{1}H-NMR (400 MHz, DMSO-d_6, δ ppm, *J* Hz): 2.82 (s, 3H), 3.74 (s, 3H), 6.91 (m, 2H), 7.44 (m, 1H), 7.63 (m, 3H), 7.74 (m, 1H), 8.23 (dd, *J* = 8.1, *J* = 1.1, 1H), 12.37 (s, 1H); ^{13}C-NMR (100 MHz, DMSO-d_6, δ ppm): 21.7, 55.6, 111.8, 114.4, 118.6, 121.4, 125.0, 125.2, 125.9, 133.0, 133.1, 138.6, 155.5, 155.9, 164.3, 176.6; HRMS *m/z* (ES+): calcd. for $C_{18}H_{16}N_2NaO_3^+$ [M+Na]$^+$ 331.1053, found 331.1042

*2-Methyl-4-oxo-1,4-dihydroquinoline-3-carboxylic acid benzylamide (**5i**):* Yield 0.231 g (79%), m.p. 250 °C; ^{1}H-NMR (400 MHz, DMSO-d_6, δ ppm, *J* Hz): 2.79 (s, 3H), 4.51 (d, *J* = 5.7, 2H), 7.25 (m, 1H), 7.37 (m, 5H), 7.61 (m, 1H), 7.71 (m, 1H), 8.18 (dd, *J* = 8.1, *J* = 1.3, 1H), 10.55 (t, *J* = 5.7, 1H); ^{13}C-NMR (100 MHz, DMSO-d_6, δ ppm): 21.5, 42.6, 111.7, 118.5, 124.7, 125.3, 125.8, 127.1, 127.7, 128.8, 132.9, 138.8, 140.3, 155.5, 166.5, 176.6; HRMS *m/z* (ES+): calcd. for $C_{18}H_{16}N_2NaO_2^+$ [M+Na]$^+$ 315.1104, found 315.1080

*2-Propyl-1H-quinolin-4-one (**8a**):* Yield 0.135 g (72%), m.p. 158 °C; ^{1}H-NMR (400 MHz, DMSO-d_6, δ ppm, *J* Hz): 0.93 (t, *J* = 7.3, 3H), 1.70 (sext, *J* = 7.4, 2H), 2.56 (t, *J* = 7.6, 2H), 5.93 (s, 1H), 7.27 (m, 1H), 7.53 (m, 1H), 7.61 (m, 1H), 8.05 (dd, *J* = 8.0, *J* = 1.4, 1H), 11.51 (br s, 1H); ^{13}C-NMR (100 MHz, DMSO-d_6, δ ppm): 13.9, 22.1, 35.6, 108.2, 118.4, 123.2, 125.1, 125.2, 131.9, 140.6, 153.8, 177.3; HRMS *m/z* (ES+): calcd. for $C_{12}H_{14}NO^+$ [M+H]$^+$ 188.1070, found 188.1068

*2-Isobutyl-1H-quinolin-4-one (**8b**):* Yield 0.150 g (74%), m.p. 198 °C; ^{1}H-NMR (400 MHz, DMSO-d_6, δ ppm, *J* Hz): 0.92 (d, *J* = 6.6, 6H), 2.05 (m, 1H), 2.45 (d, *J* = 7.4, 2H), 5.91 (s, 1H), 7.27 (m, 1H), 7.54 (m, 1H), 7.61 (m, 1H), 8.05 (dd, *J* = 8.1, *J* = 1.4, 1H), 11.48 (br s, 1H); ^{13}C-NMR (100 MHz, DMSO-d_6, δ ppm): 22.5, 28.3, 42.8, 109.0, 118.4, 123.2, 125.1, 125.2, 131.9, 140.6, 152.9, 177.2; HRMS *m/z* (ES+): calcd. for $C_{13}H_{16}NO^+$ [M+H]$^+$ 202.1226, found 202.1221

*2-Pentyl-1H-quinolin-4-one (**8c**):* Yield 0.194 g (90%), m.p. 142 °C; ^{1}H-NMR (400 MHz, DMSO-d_6, δ ppm, *J* Hz): 0.87 (t, *J* = 6.6, 3H), 1.31 (m, 4H), 1.67 (m, 2H), 2.58 (m, 2H), 5.93 (s, 1H), 7.27 (m, 1H), 7.55 (m, 1H), 7.61 (m, 1H), 8.05 (m, 1H), 11.51 (br s, 1H); ^{13}C-NMR (100 MHz, DMSO-d_6, δ ppm): 14.3, 22.3, 28.5, 31.2, 33.7, 108.1, 118.3, 123.2, 125.1, 125.2, 131.9, 140.6, 154.0, 177.3; HRMS *m/z* (ES+): calcd. for $C_{14}H_{18}NO^+$ [M+H]$^+$ 216.1383, found 216.1389

*2-Heptyl-1H-quinolin-4-one (**8d**):* Yield 0.219 g (90%), m.p. 144 °C; ^{1}H-NMR (400 MHz, DMSO-d_6, δ ppm, *J* Hz): 0.84 (t, *J* = 6.8, 3H), 1.27 (m, 8H), 1.66 (m, 2H), 2.58 (m, 2H), 5.92 (s, 1H), 7.27 (m, 1H), 7.54 (m, 1H), 7.61 (m, 1H), 8.05 (m, 1H), 11.50 (br s, 1H); ^{13}C-NMR (100 MHz, DMSO-d_6, δ ppm): 14.4, 22.5, 28.8, 28.9, 29.0, 31.6, 33.7, 108.1, 118.3, 123.2, 125.1, 125.2, 131.9, 140.6, 154.0, 177.3; HRMS *m/z* (ES+): calcd. for $C_{16}H_{22}NO^+$ [M+H]$^+$ 244.1696, found 244.1680

Synthesis of *N*-hydroxy derivatives (4), general procedure:

To the corresponding nitro-intermediate **3** (100 mg) in CH_3OH (10–15 mL) was added $HCOONH_4$ (300 mg) and Pd on charcoal (10 mg, 10 wt % Pd). The mixture was magnetically stirred for 90 min at rt and was then suction-filtered through a pad of celite on a sintered glass funnel. The celite was rinsed thoroughly with methanol and the solvent was removed from the filtrate under reduced pressure. Then, water (50 mL) was added to the solid residue and the product was extracted in CH_2Cl_2 (3 × 20 mL). The combined organic layers were dried with anhydrous sodium sulfate, the drying agent was filtered off and the solvent was removed under reduced pressure. The crude products were purified by column chromatography on silica gel with Et_2O as the eluent, increasing polarity to Et_2O/MeOH 20:1, where necessary.

*1-Hydroxy-4-oxo-2-propyl-1,4-dihydroquinoline-3-carboxylic acid phenylamide (**4a**):* Yield 0.048 g (57%), m.p. 203 °C; ^{1}H-NMR (400 MHz, DMSO-d_6, δ ppm, *J* Hz): 1.00 (t, *J* = 7.4, 3H), 1.79 (m, 2H), 3.12 (m, 2H), 7.07 (m, 1H), 7.34 (m, 2H), 7.49 (m, 1H), 7.71 (m, 2H), 7.84 (m, 1H), 7.94 (m, 1H), 8.26 (dd, *J* = 8.1, *J* = 1.2, 1H), 11.34 (s, 1H), 12.12 (br s, 1H); ^{13}C-NMR (100 MHz, DMSO-d_6, δ ppm): 14.7, 22.2, 31.3, 115.0, 115.7, 120.0, 123.7, 125.0, 125.5, 126.0, 129.2, 133.4, 139.8, 139.9, 155.6, 164.6, 173.7; HRMS *m/z* (ES+): calcd. for $C_{19}H_{18}N_2NaO_3^+$ [M+Na]$^+$ 345.1210, found 345.1217

*1-Hydroxy-2-isobutyl-4-oxo-1,4-dihydroquinoline-3-carboxylic acid phenylamide (**4b**):* Yield 0.064 g (75%), m.p. 231 °C; ^{1}H-NMR (400 MHz, DMSO-d_6, δ ppm, *J* Hz): 0.95 (d, *J* = 6.7, 6H), 2.22 (m, 1H), 3.13 (d, *J* = 7.2, 2H), 7.07 (m, 1H), 7.34 (m, 2H), 7.48 (m, 1H), 7.70 (m, 2H), 7.82 (m, 1H), 7.92 (m, 1H), 8.25 (dd, *J* = 8.1, *J* = 1.1, 1H), 11.11 (s, 1H), 12.07 (br s, 1H); ^{13}C-NMR (100 MHz, DMSO-d_6, δ ppm): 22.9, 29.4, 36.9, 115.7, 116.3, 119.9, 123.6, 124.9, 125.5, 126.0, 129.2, 133.3, 139.9, 140.0, 154.3, 164.8, 173.4; HRMS *m/z* (ES-): calcd. for $C_{20}H_{19}N_2O_3^-$ [M-H]$^-$ 335.1401, found 335.1389

*1-Hydroxy-4-oxo-2-pentyl-1,4-dihydroquinoline-3-carboxylic acid phenylamide (**4c**):* Yield 0.052 g (60%), m.p. 218 °C; ^{1}H-NMR (400 MHz, DMSO-d_6, δ ppm, *J* Hz): 0.86 (t, *J* = 7.2, 3H), 1.36 (m, 4H), 1.78 (m, 2H), 3.12 (m, 2H), 7.07 (m, 1H), 7.34 (m, 2H), 7.49 (m, 1H), 7.71 (m, 2H), 7.84 (m, 1H), 7.93 (m, 1H), 8.25 (dd, *J* = 8.1, *J* = 1.1, 1H), 11.31 (s, 1H), 12.10 (br s, 1H); ^{13}C-NMR (100 MHz, DMSO-d_6, δ ppm): 14.2, 22.1, 28.2, 29.3, 31.8, 115.0, 115.7, 119.9, 123.6, 125.0, 125.5, 126.0, 129.2, 133.4, 139.8, 139.9, 155.8, 164.6, 173.7; HRMS *m/z* (ES-): calcd. for $C_{21}H_{21}N_2O_3^-$ [M-H]$^-$ 349.1558, found 349.1572

*2-Heptyl-1-hydroxy-4-oxo-1,4-dihydroquinoline-3-carboxylic acid phenylamide (**4d**):* Yield 0.060 g (70%), m.p. 188 °C; ^{1}H-NMR (400 MHz, DMSO-d_6, δ ppm, *J* Hz): 0.82 (t, *J* = 7.0, 3H), 1.27 (m, 6H), 1.40 (m, 2H), 1.77 (m, 2H), 3.11 (m, 2H), 7.07 (m, 1H), 7.34 (m, 2H), 7.49 (m, 1H), 7.71 (m, 2H), 7.84 (m, 1H), 7.93 (m, 1H), 8.25 (dd, *J* = 8.1, *J* = 1.2, 1H), 11.29 (s, 1H), 12.09 (br s, 1H); ^{13}C-NMR (100 MHz, DMSO-d_6, δ ppm): 14.4, 22.5, 28.5, 28.7, 29.3, 29.6, 31.5, 115.1, 115.7, 119.9, 123.6, 125.0, 125.5, 126.0, 129.2, 133.3, 139.9, 140.0, 155.7, 164.6, 173.6; HRMS *m/z* (ES-): calcd. for $C_{23}H_{25}N_2O_3^-$ [M-H]$^-$ 377.1871, found 377.1857

*2-Heptyl-1-hydroxy-4-oxo-1,4-dihydroquinoline-3-carboxylic acid (4-methoxyphenyl)-amide (**4e**):* Yield 0.056 g (64%), m.p. 140 °C; ^{1}H-NMR (400 MHz, DMSO-d_6, δ ppm, *J* Hz): 0.82 (t, *J* = 6.9, 3H), 1.25 (m, 6H), 1.37 (m, 2H), 1.74 (m, 2H), 3.12 (m, 2H), 3.75 (s, 3H), 6.91 (m, 2H), 7.46 (m, 1H), 7.62 (m, 2H), 7.78 (m, 1H), 7.90 (m, 1H), 8.24 (dd, *J* = 8.1, *J* = 1.1, 1H), 11.32 (s, 1H), 12.41 (br s, 1H); ^{13}C-NMR (100 MHz, DMSO-d_6, δ ppm): 14.4, 22.5, 28.5, 28.7, 29.3, 29.6, 31.6, 55.6, 114.3, 114.6, 115.8, 121.4, 124.9, 125.6, 125.9, 133.1, 140.0, 155.6, 155.8, 164.2, 173.5; HRMS *m/z* (ES-): calcd. for $C_{24}H_{27}N_2O_4^-$ [M-H]$^-$ 407.1976, found 407.1965
