# Supplementary material for: Synthetic approach to 2-alkyl-4-quinolones and 2-alkyl-4-quinolone-3-carboxamides based on common β-keto amide precursors
Source: Beilstein J Org Chem. 2023 Nov 23;19:1804–10. doi: 10.3762/bjoc.19.132 (PMC10682542; doi:10.3762/bjoc.19.132)

BEILSTEIN JOURNAL OF ORGANIC CHEMISTRY

Supporting Information

for

Synthetic approach to 2-alkyl-4-quinolones and 2-alkyl-4-quinolone-3-carboxamides based on common β-keto amide precursors

Yordanka Mollova-Sapundzhieva, Plamen Angelov, Danail Georgiev and Pavel Yanev

Beilstein J. Org. Chem. **2023,** *19,* 1804–1810. doi:10.3762/bjoc.19.132

Processed NMR spectra

License and Terms: This is a supporting information file under the terms of the Creative Commons Attribution License (https://creativecommons.org/licenses/by/4.0). Please note that the reuse, redistribution and reproduction in particular requires that the author(s) and source are credited and that individual graphics may be subject to special legal provisions.
The license is subject to the *Beilstein Journal of Organic Chemistry* terms and conditions: (https://www.beilstein-journals.org/bjoc/terms)

Table of contents...Page

Processed NMR spectra (^{1}H/^{13}C)

1-Hydroxy-2-alkyl-4-quinolone-3-carboxamides **4** S2

2-Alkyl-4-quinolone-3-carboxamides **5** S7

2-Alkyl-4-quinolones **8** S16

Primary NMR data available at: https://doi.org/10.5281/zenodo.8337063

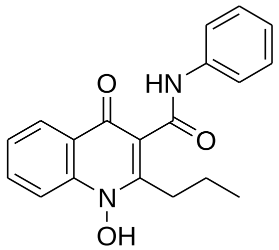

4a 400 MHz, DMSO-d_6

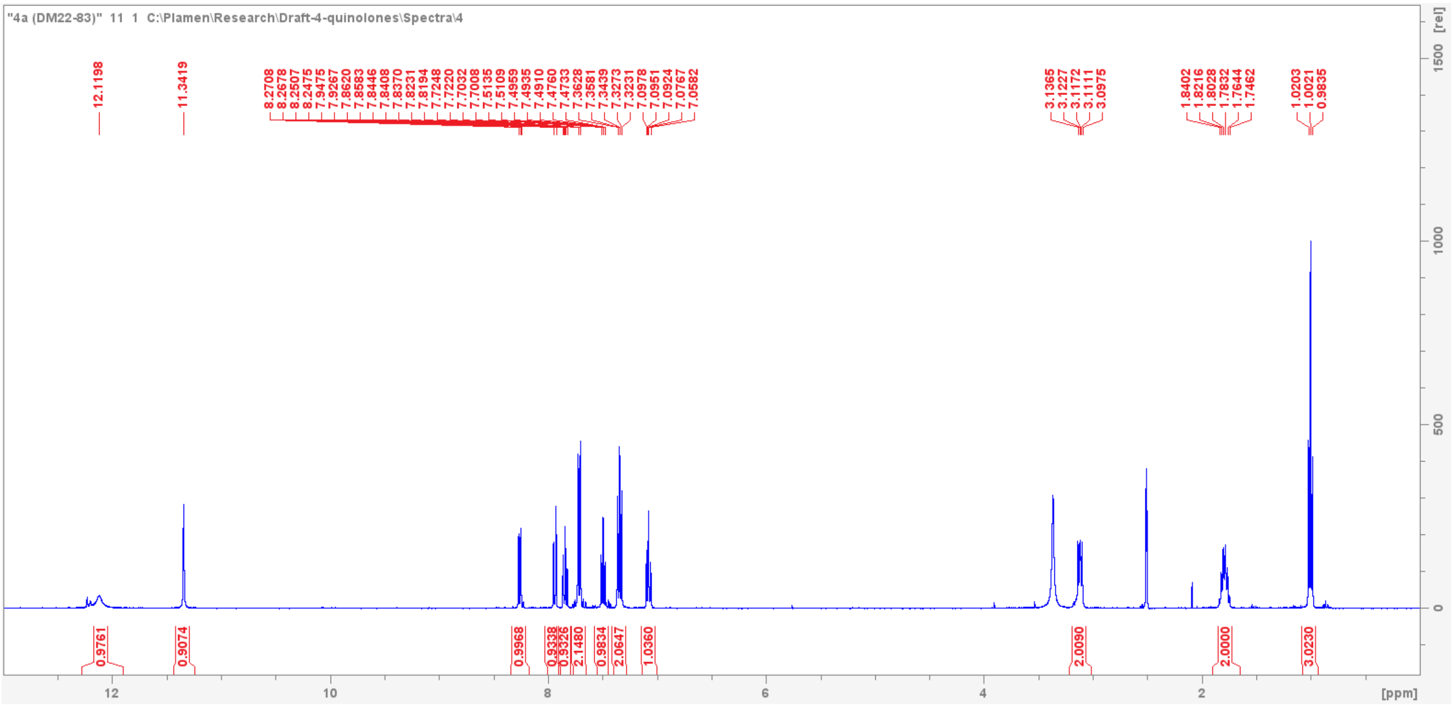

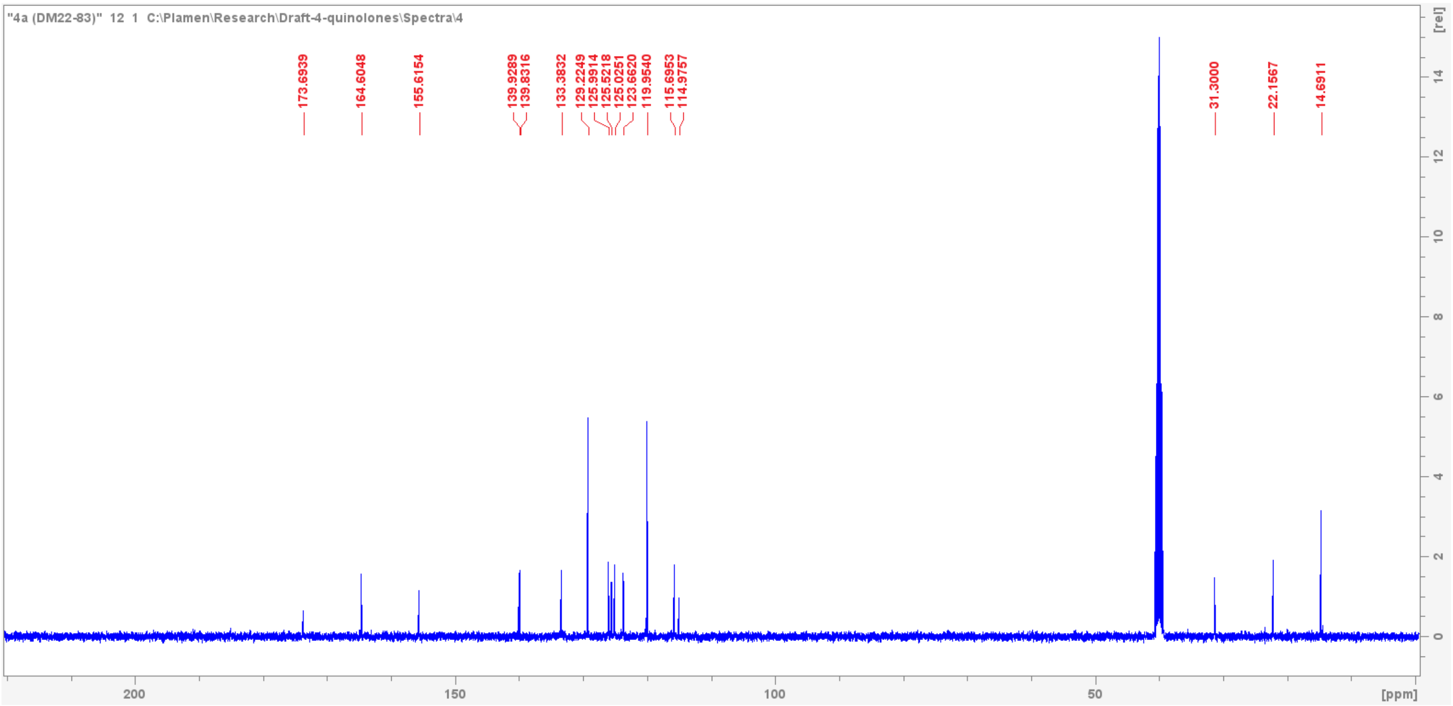

4b

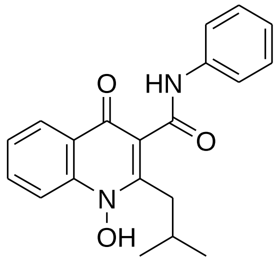

400 MHz, DMSO-d_6

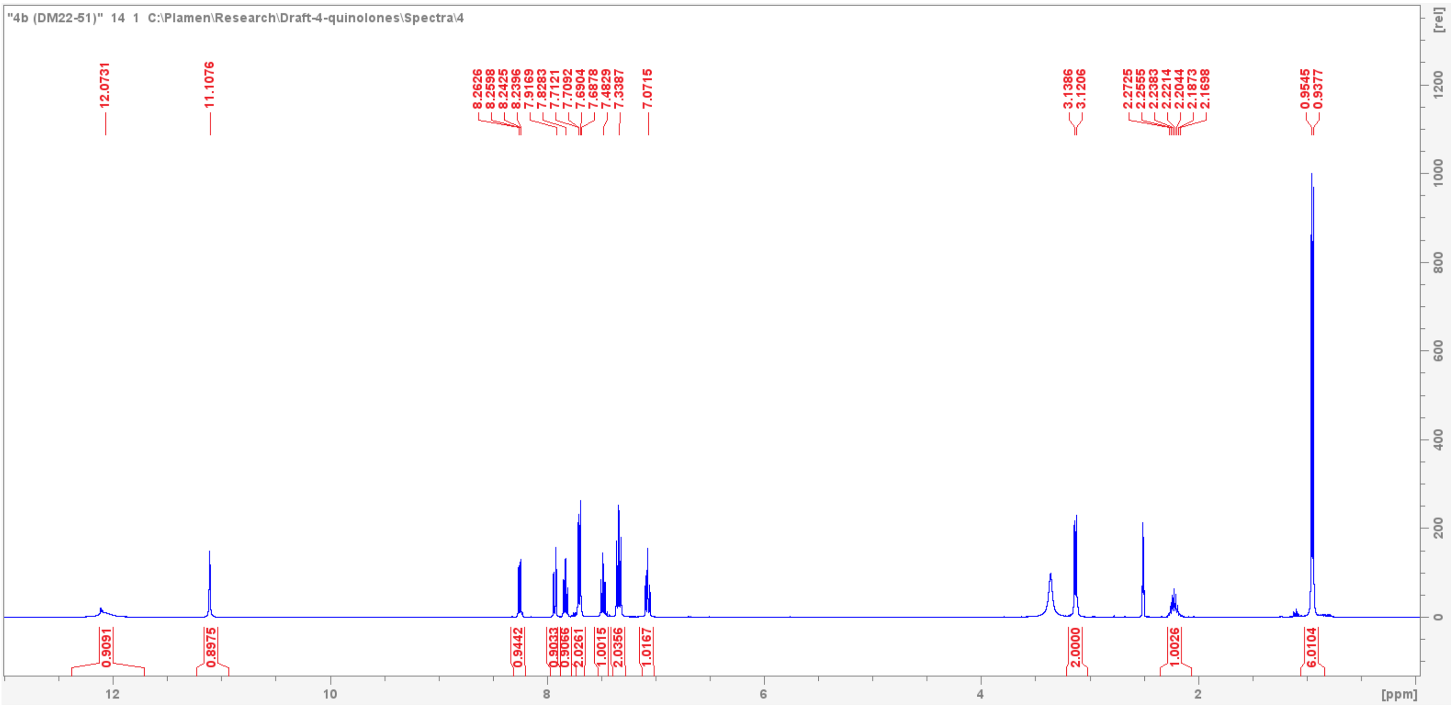

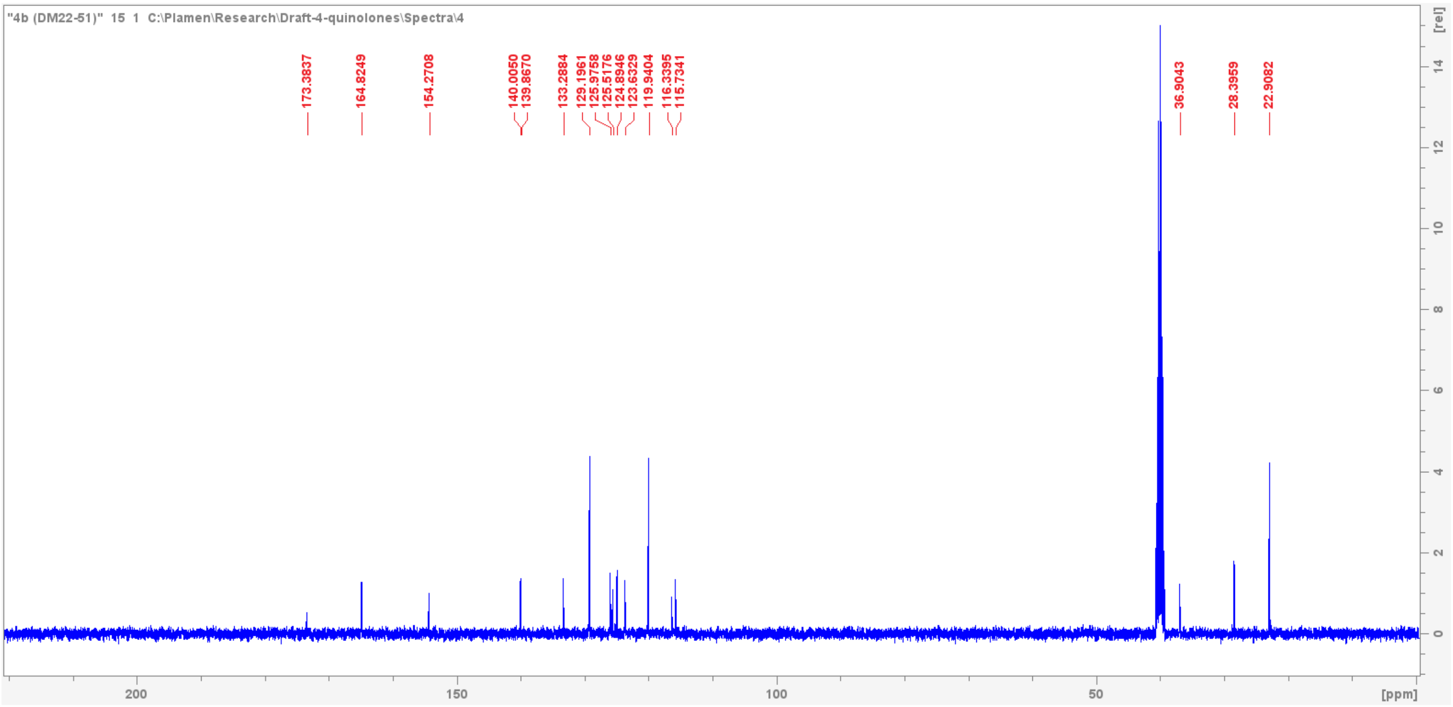

4c

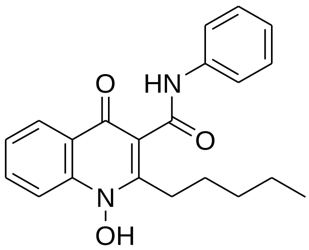

400 MHz, DMSO-d_6

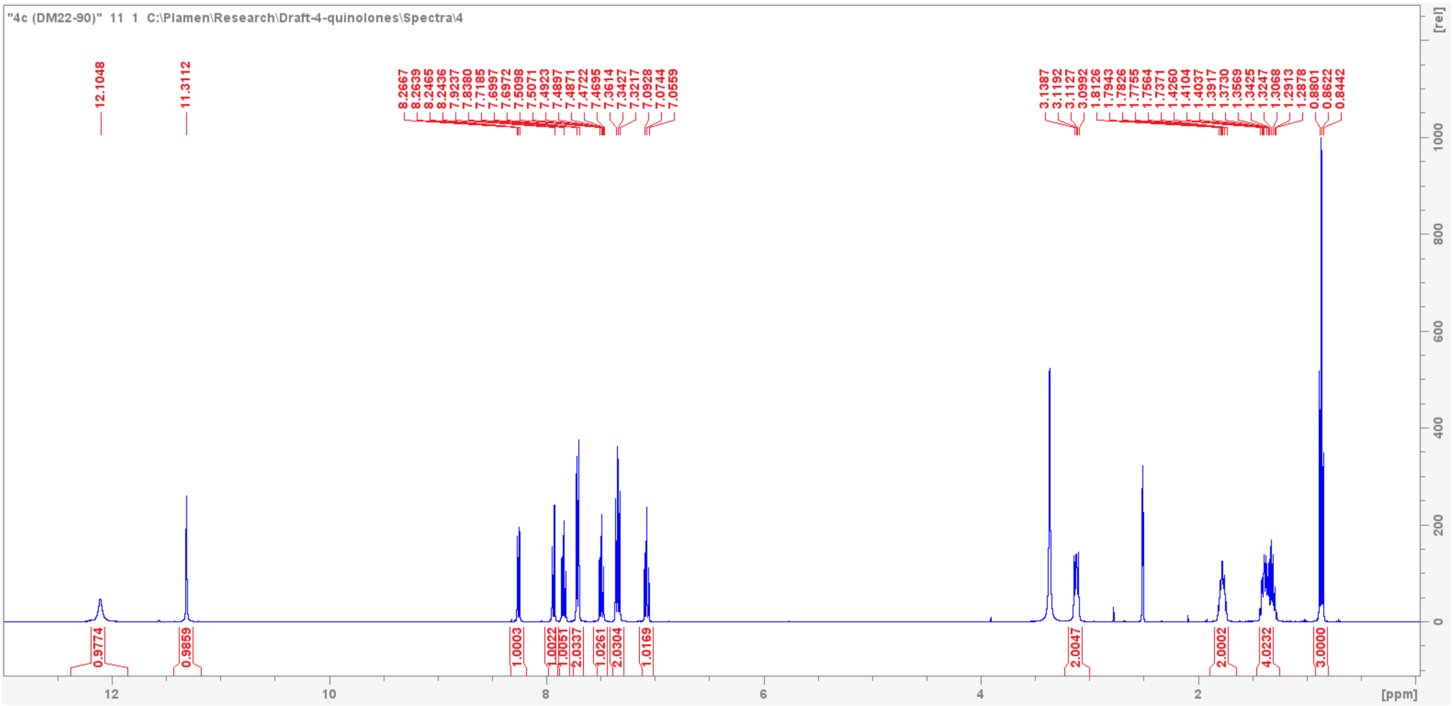

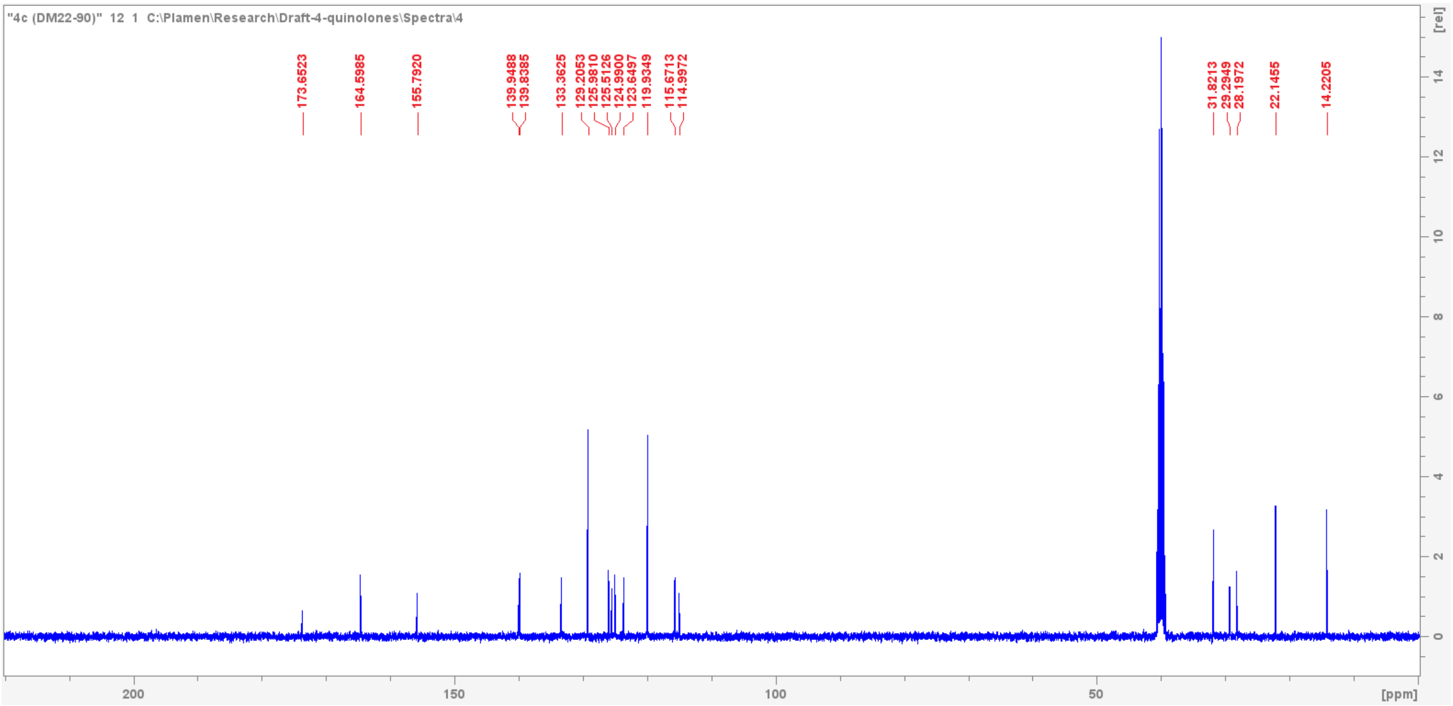

4d 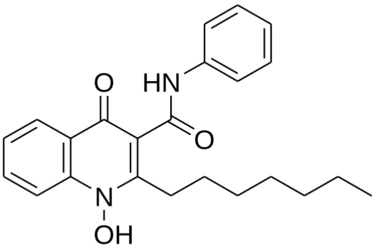

400 MHz, DMSO-d_6

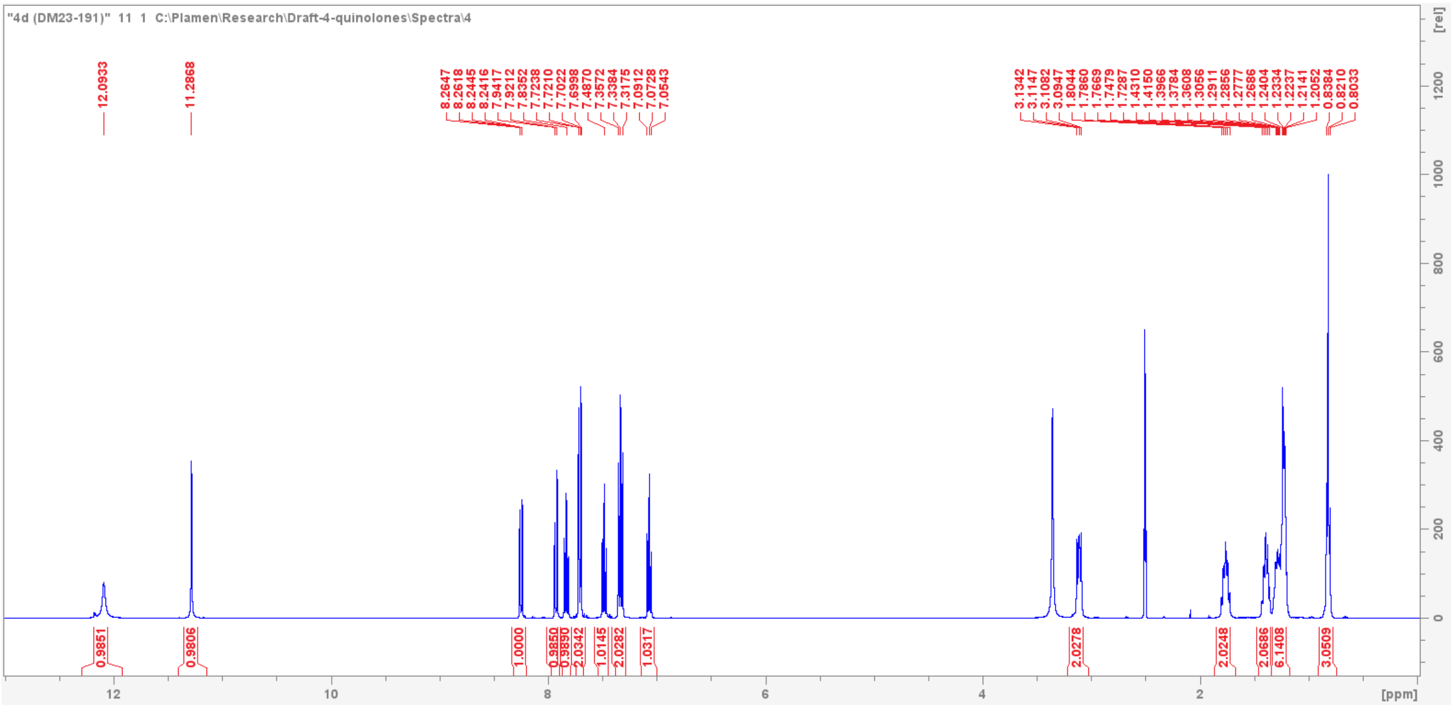

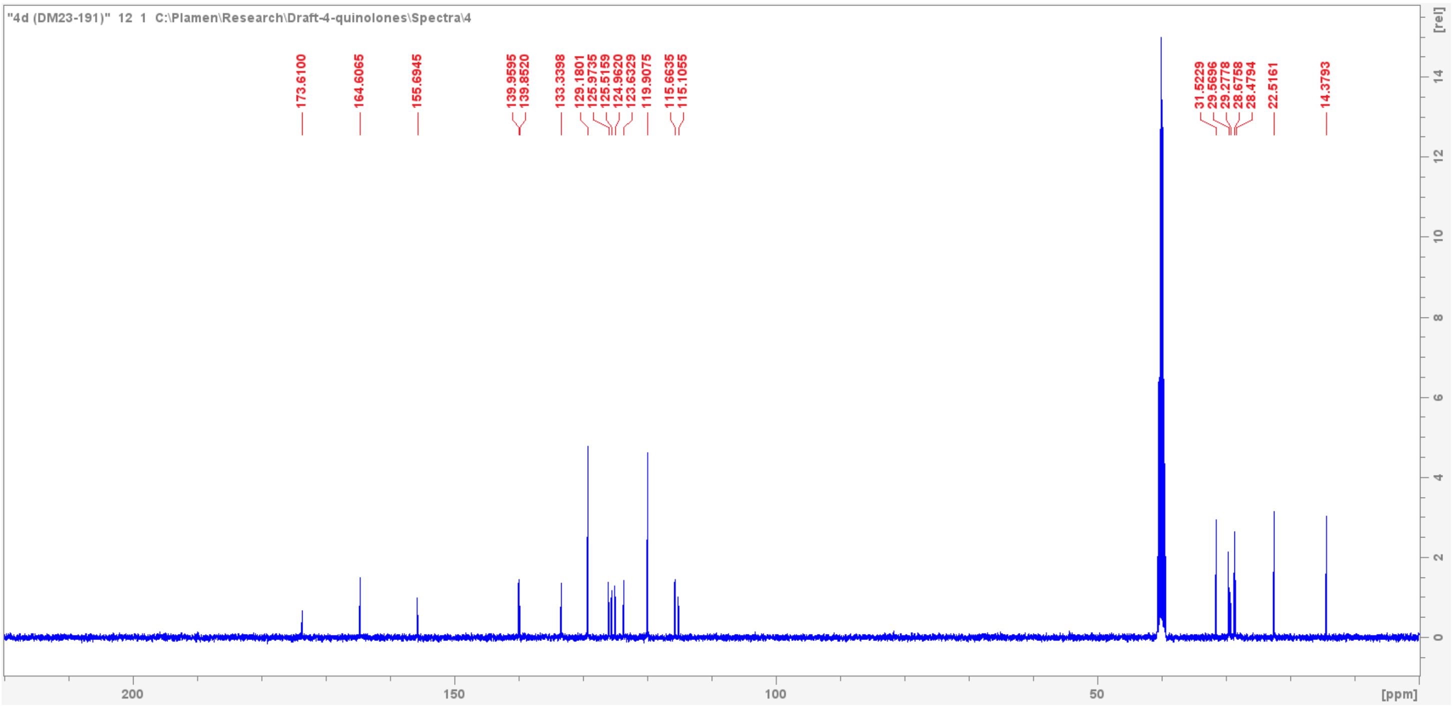

4e

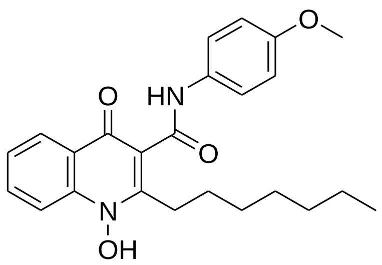

400 MHz, DMSO-d_6

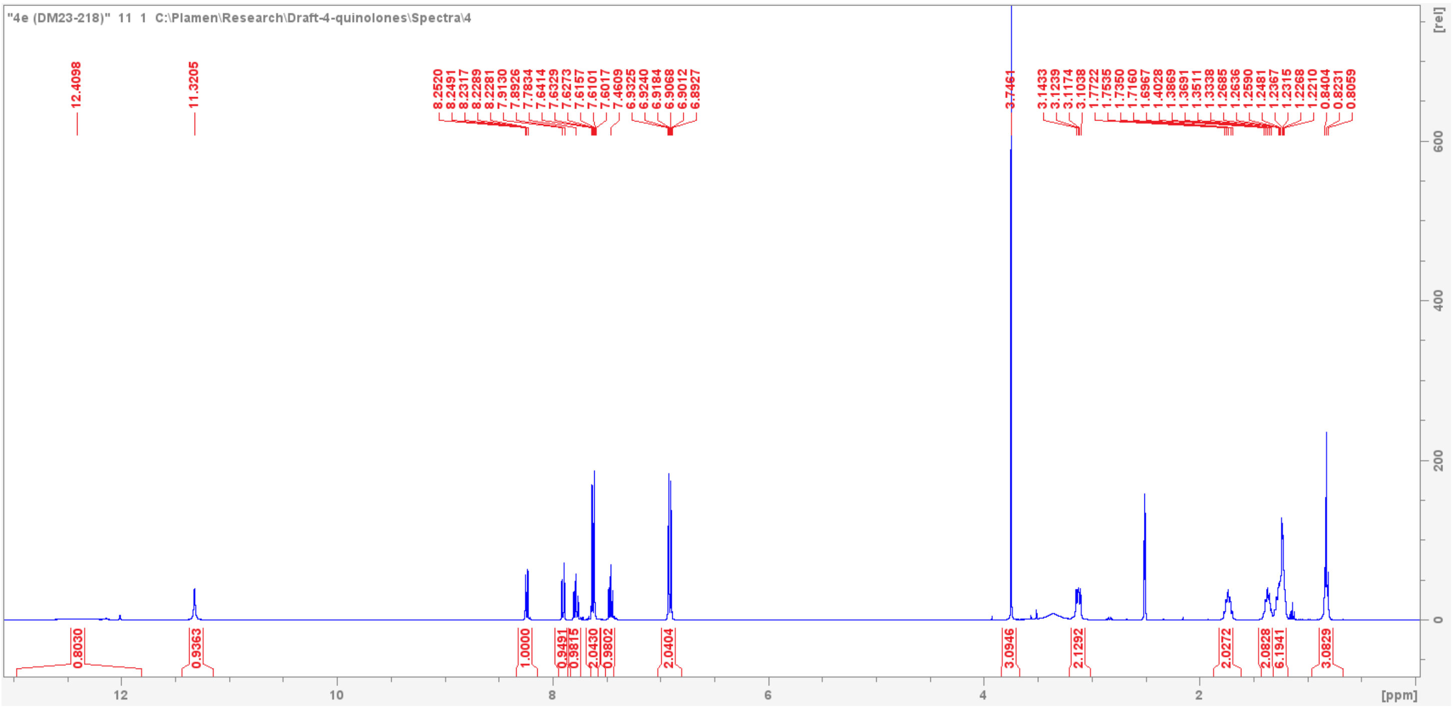

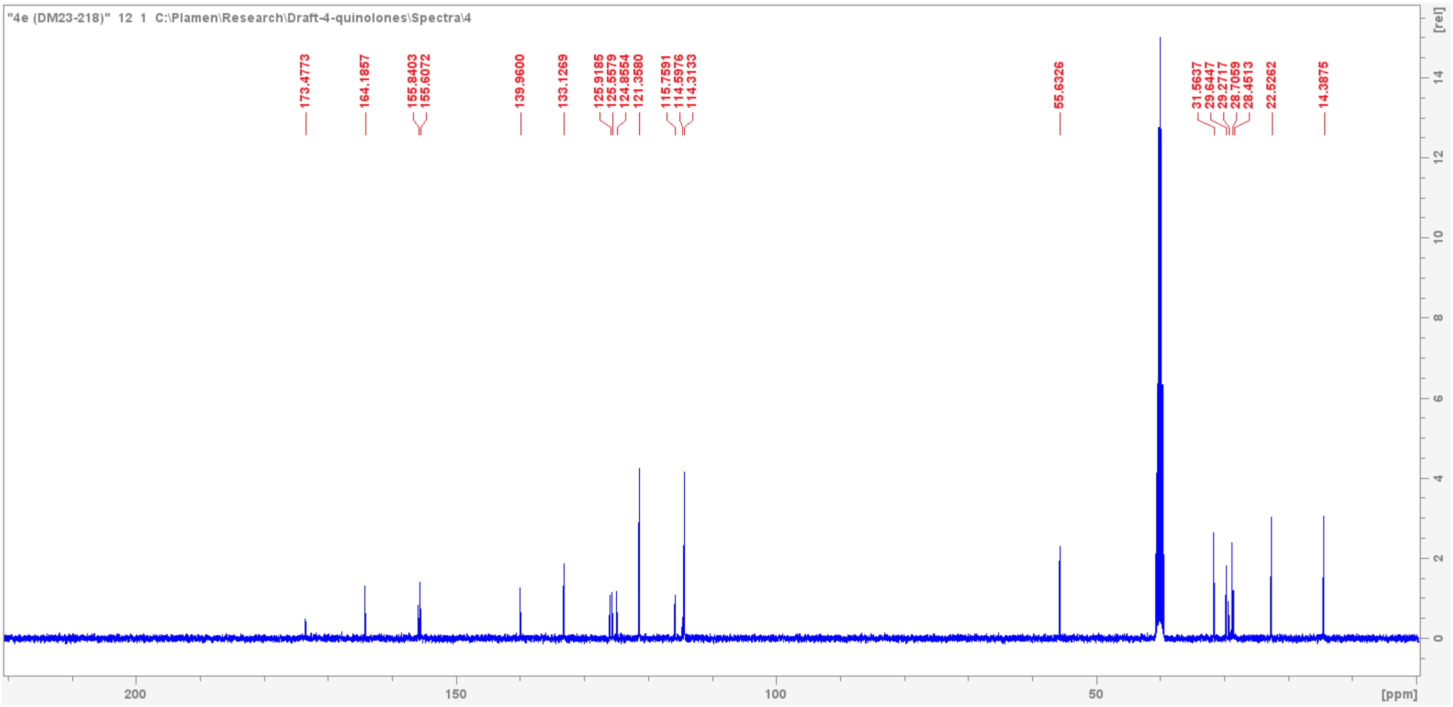

5a 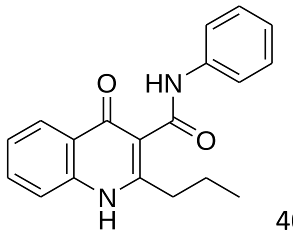

400 MHz, DMSO-d_6

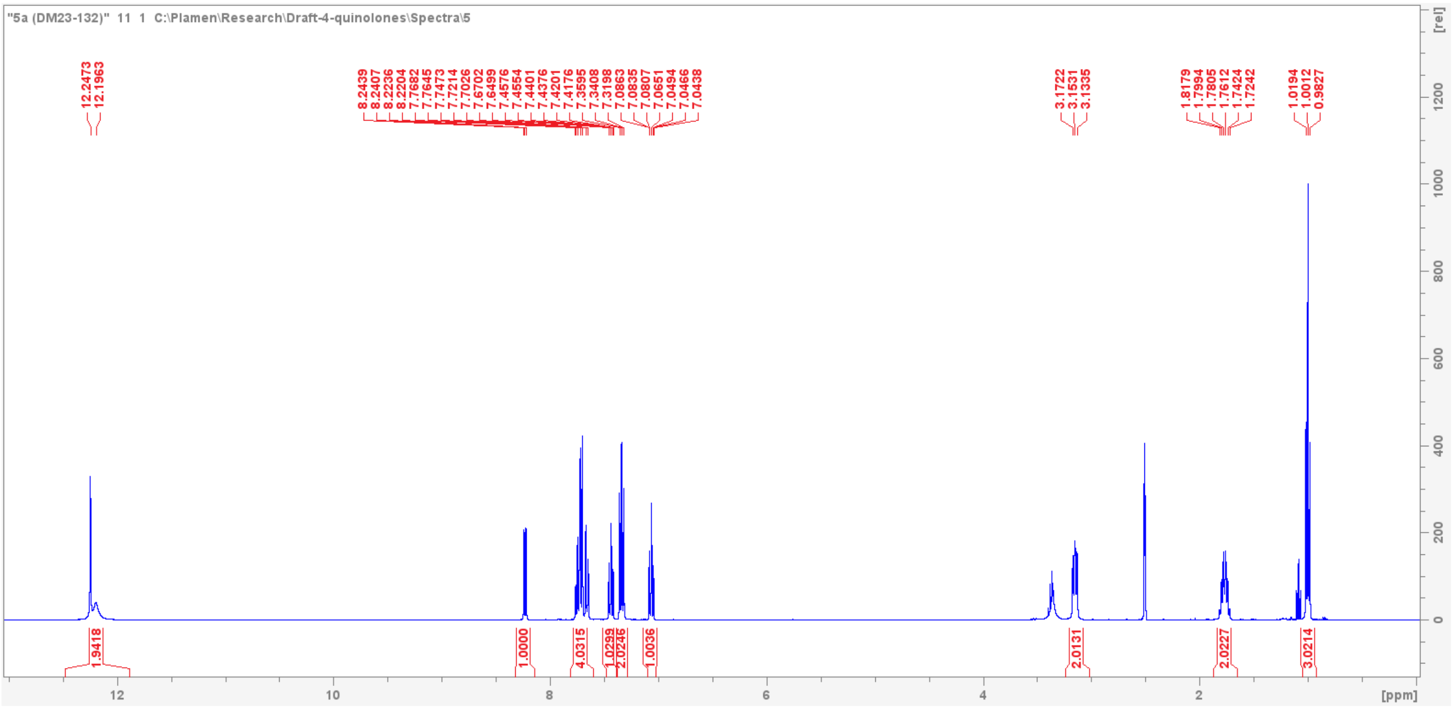

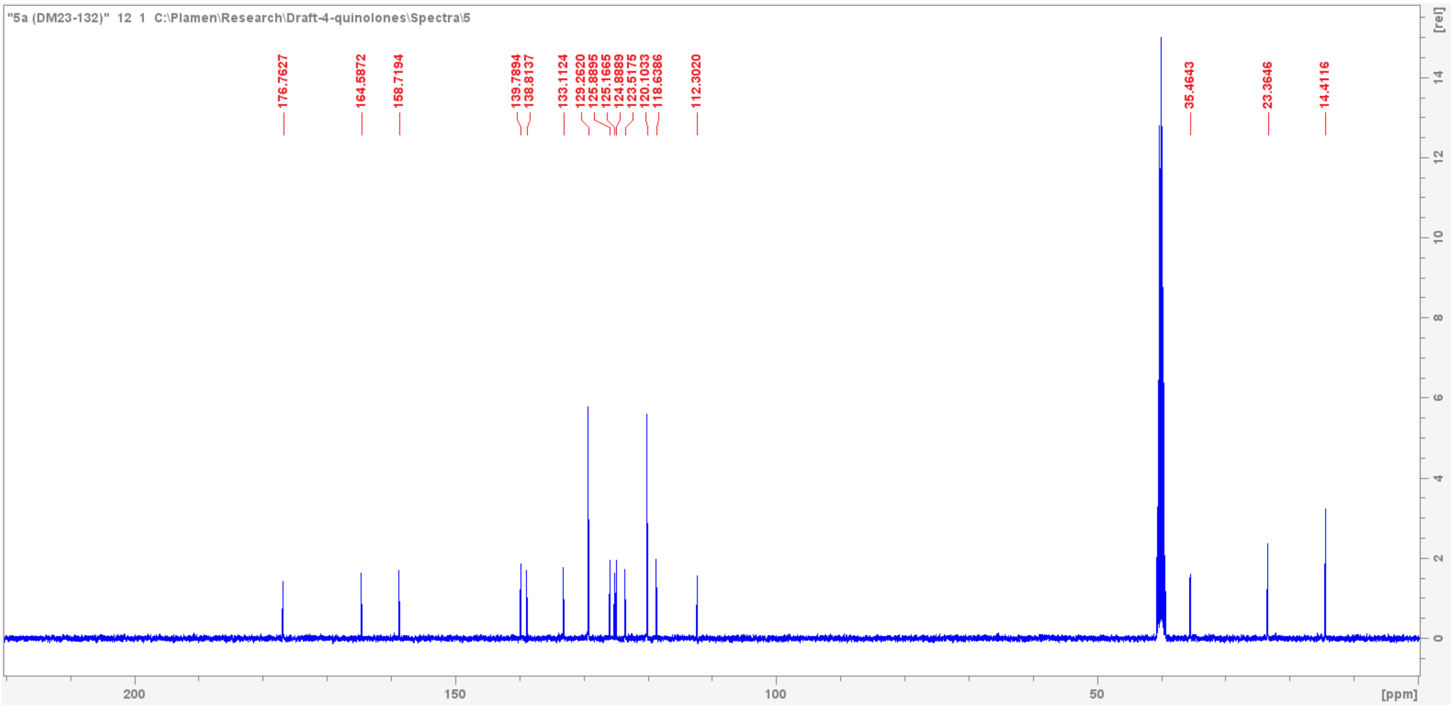

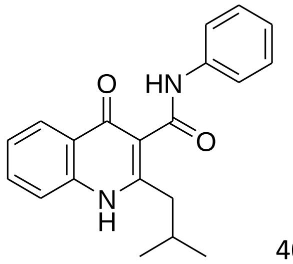

5b 400 MHz, DMSO-d_6

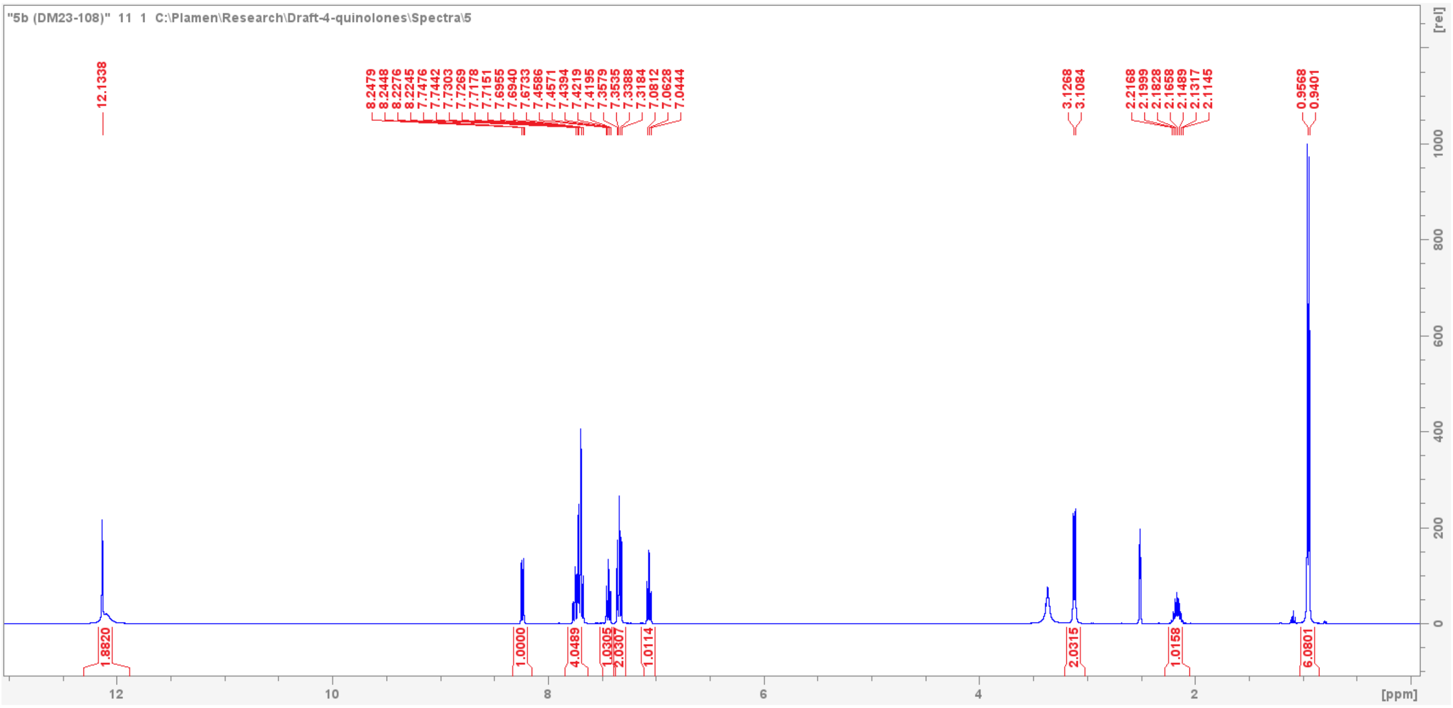

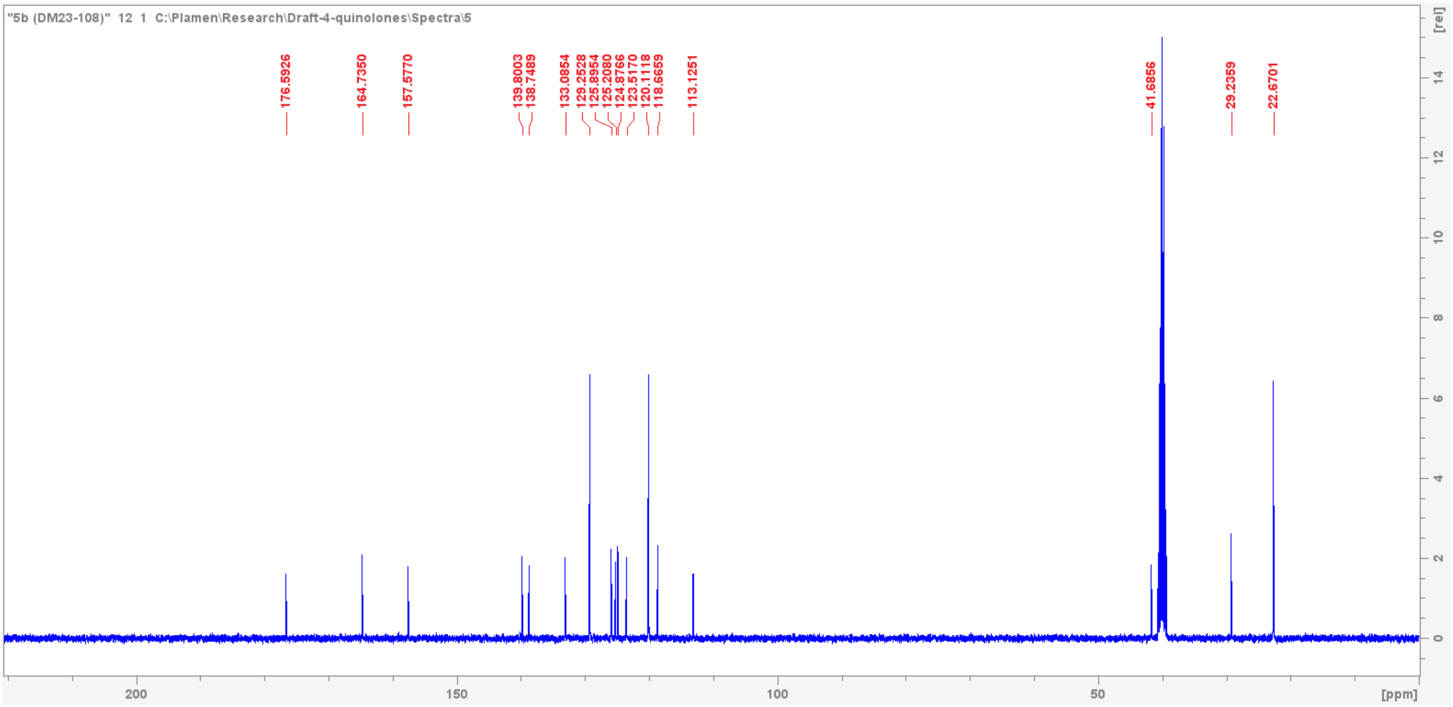

5c 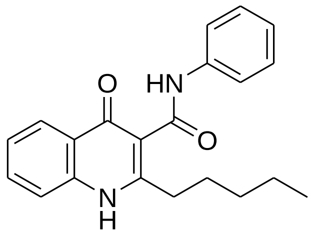

400 MHz, DMSO-d_6

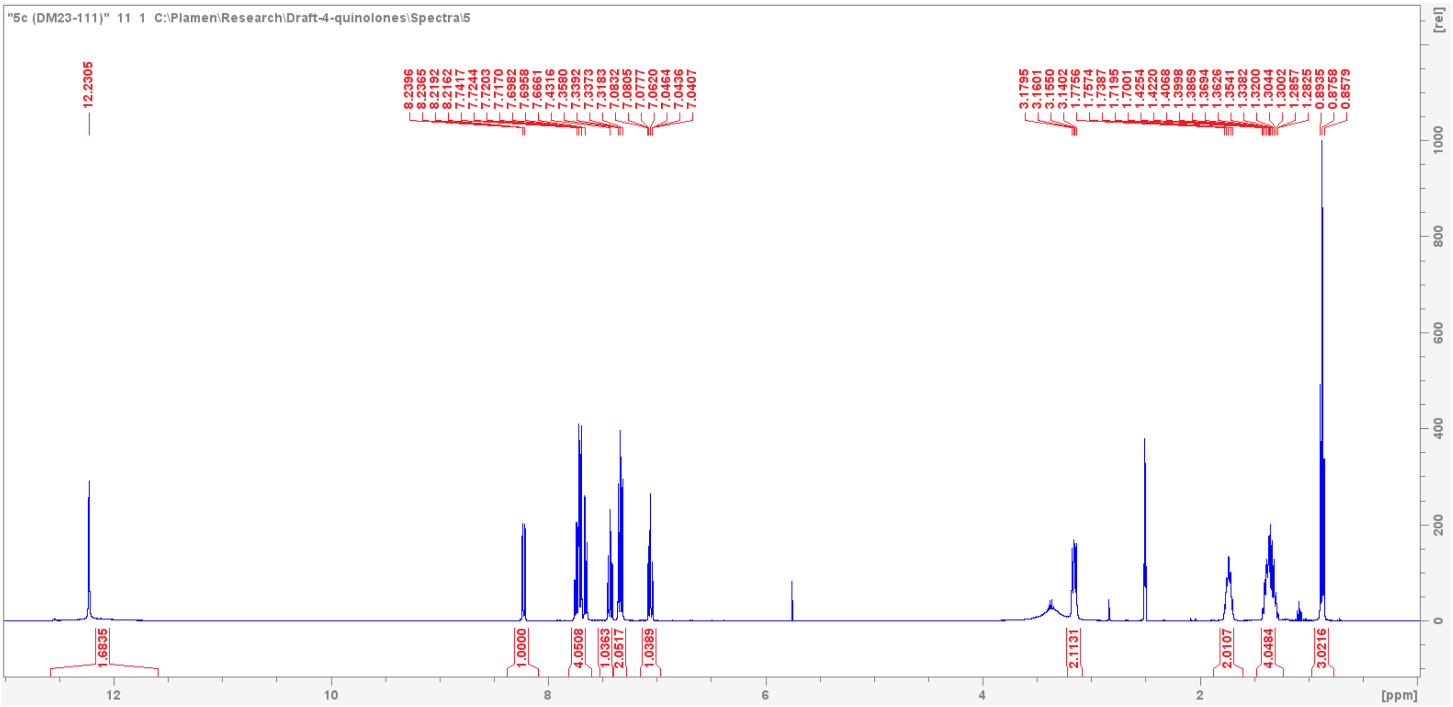

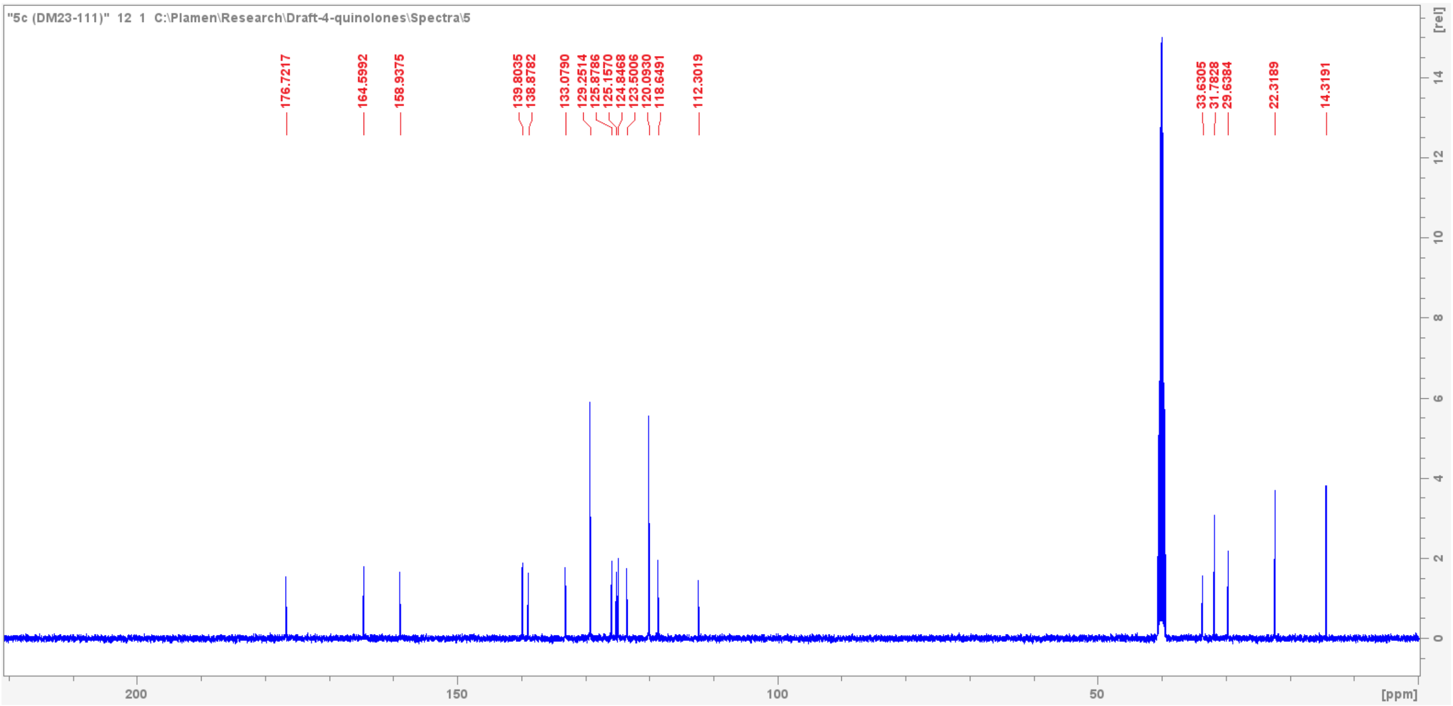

5d 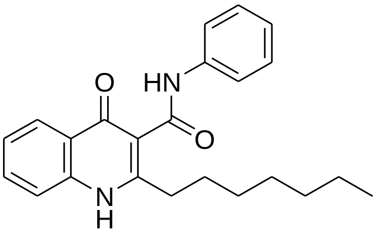

400 MHz, DMSO-d_6

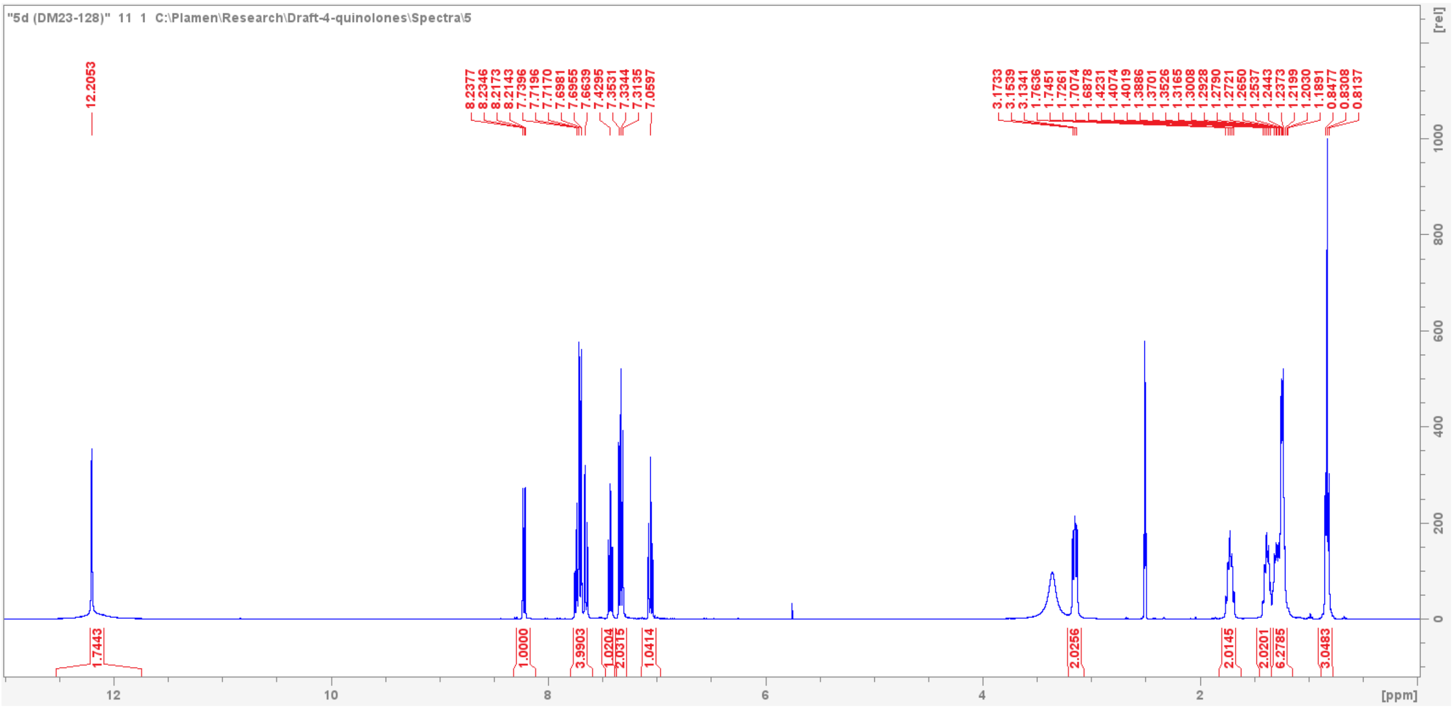

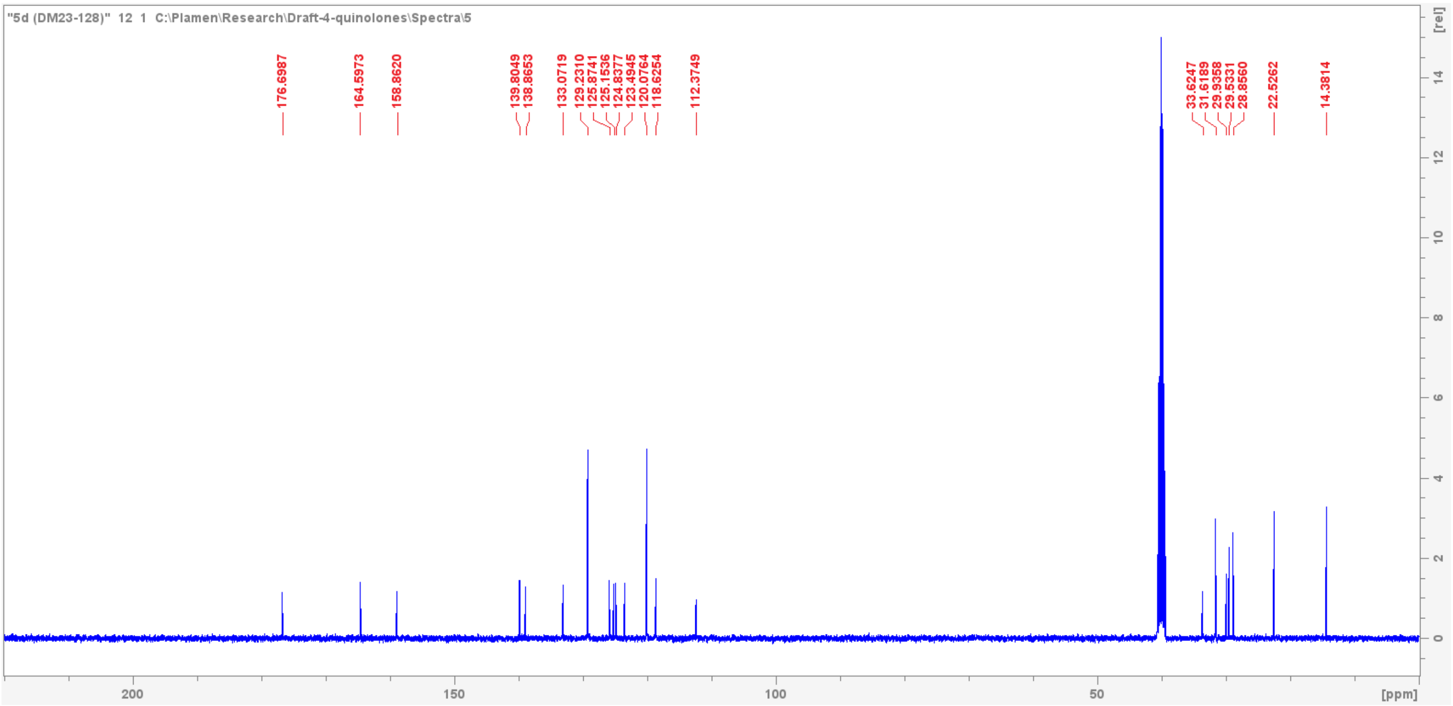

5e 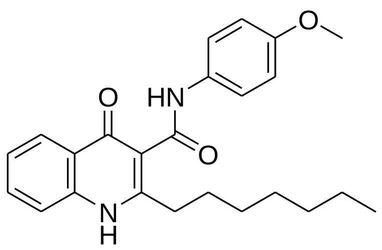

400 MHz, DMSO-d_6

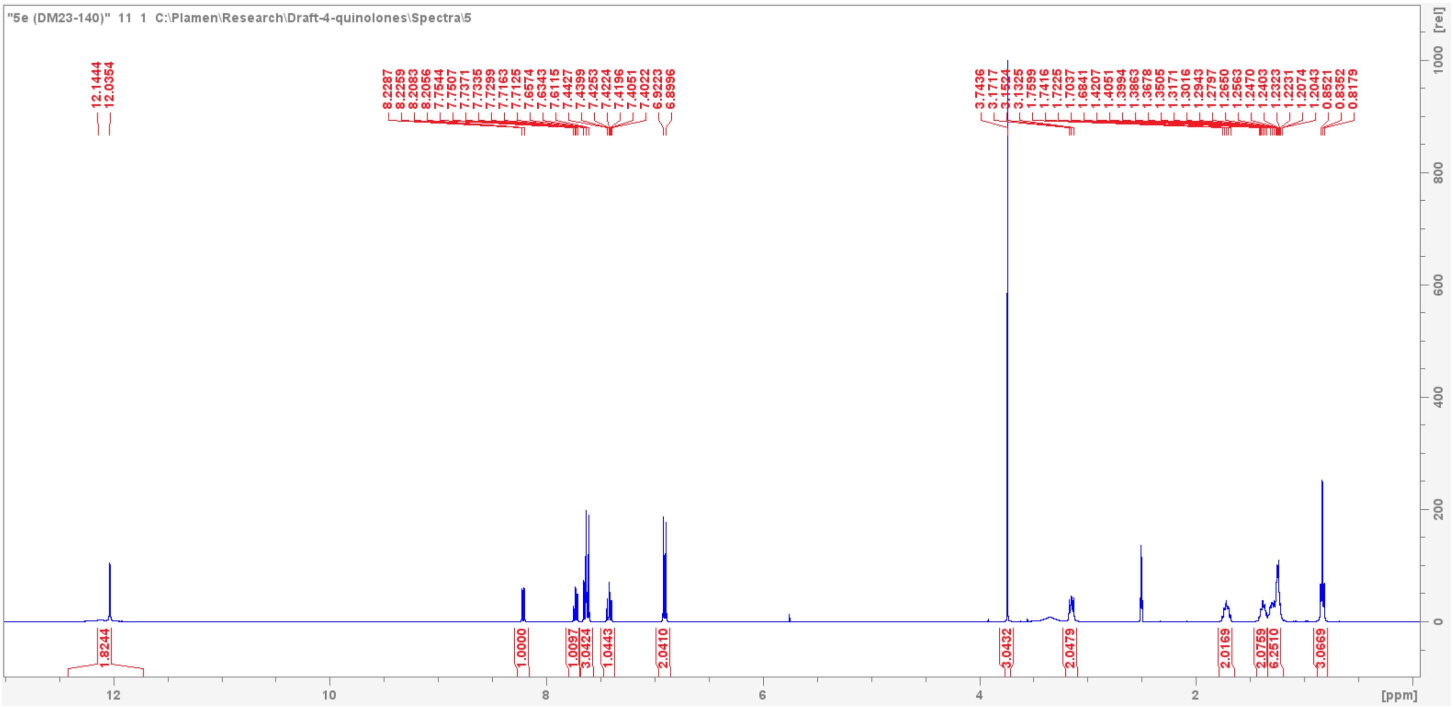

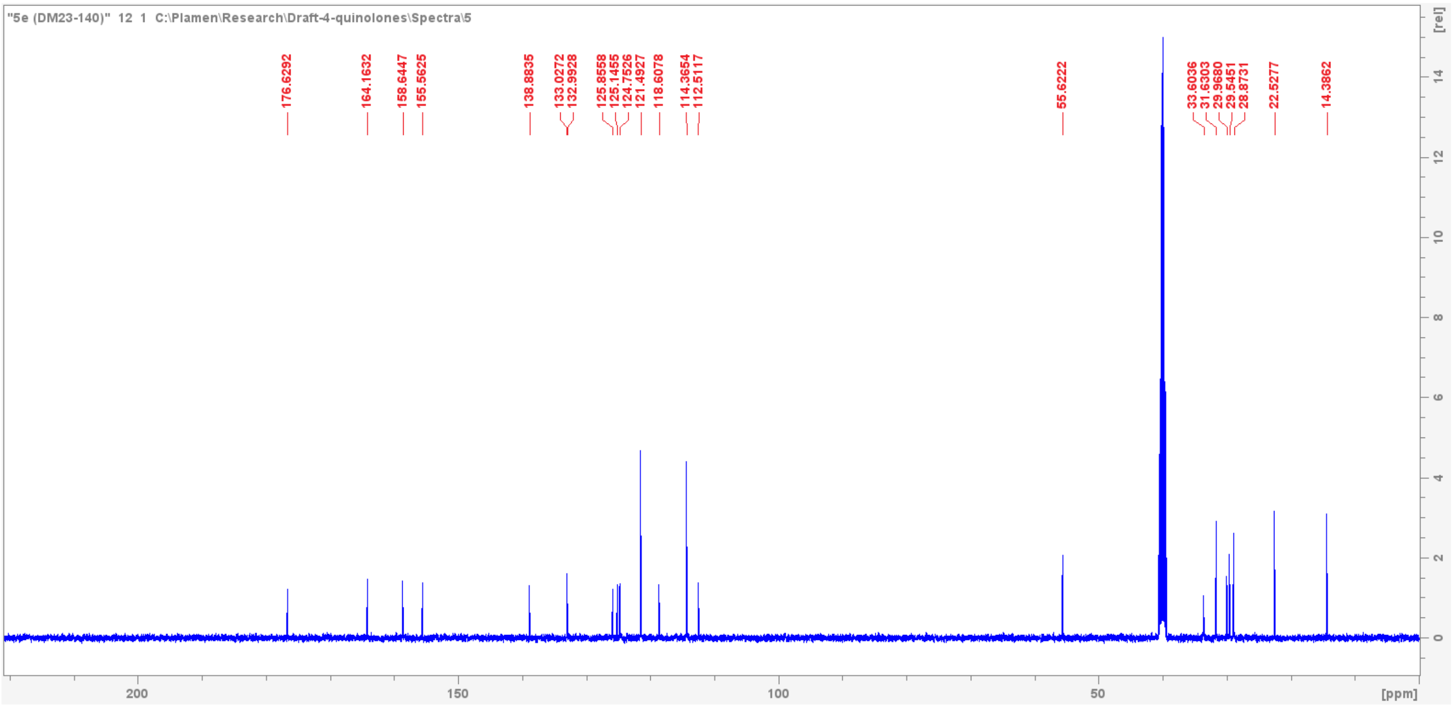

5f 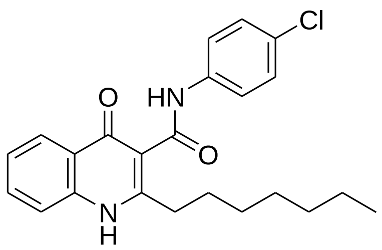

400 MHz, DMSO-d_6

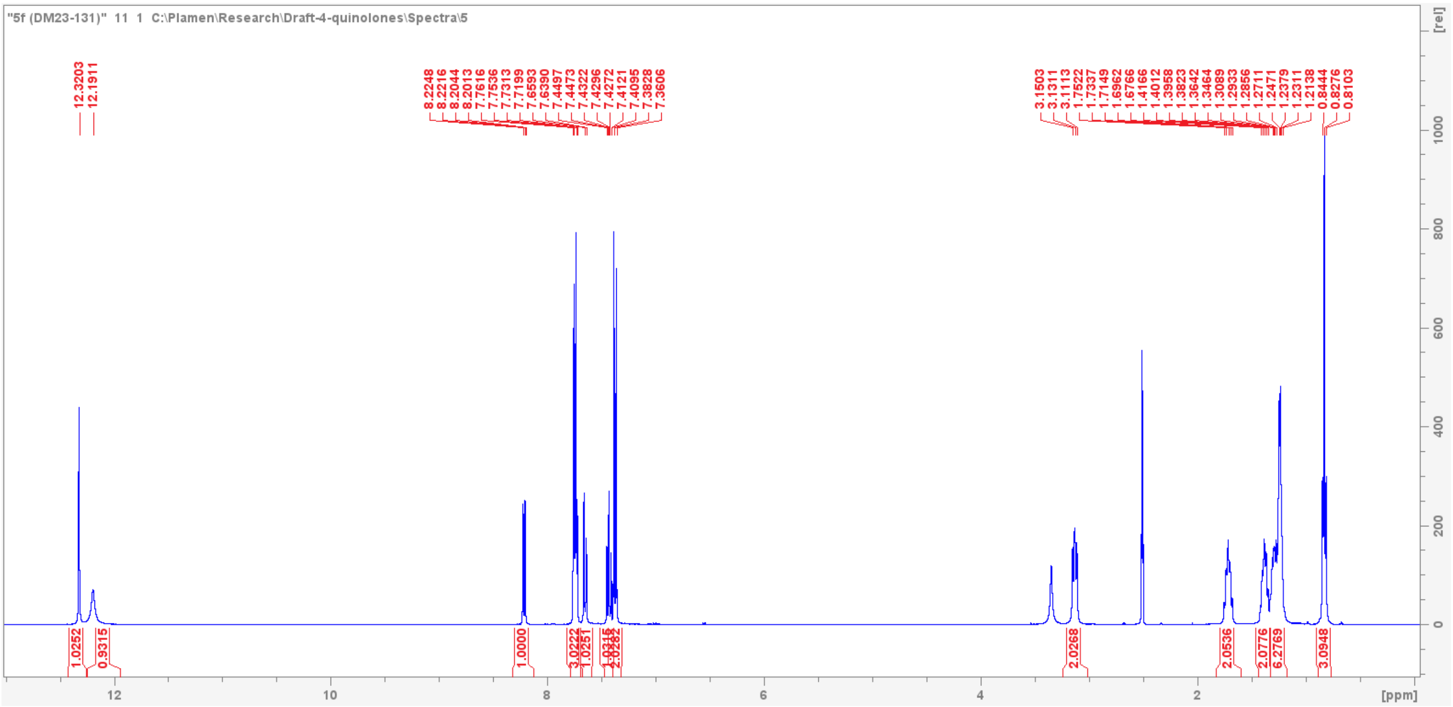

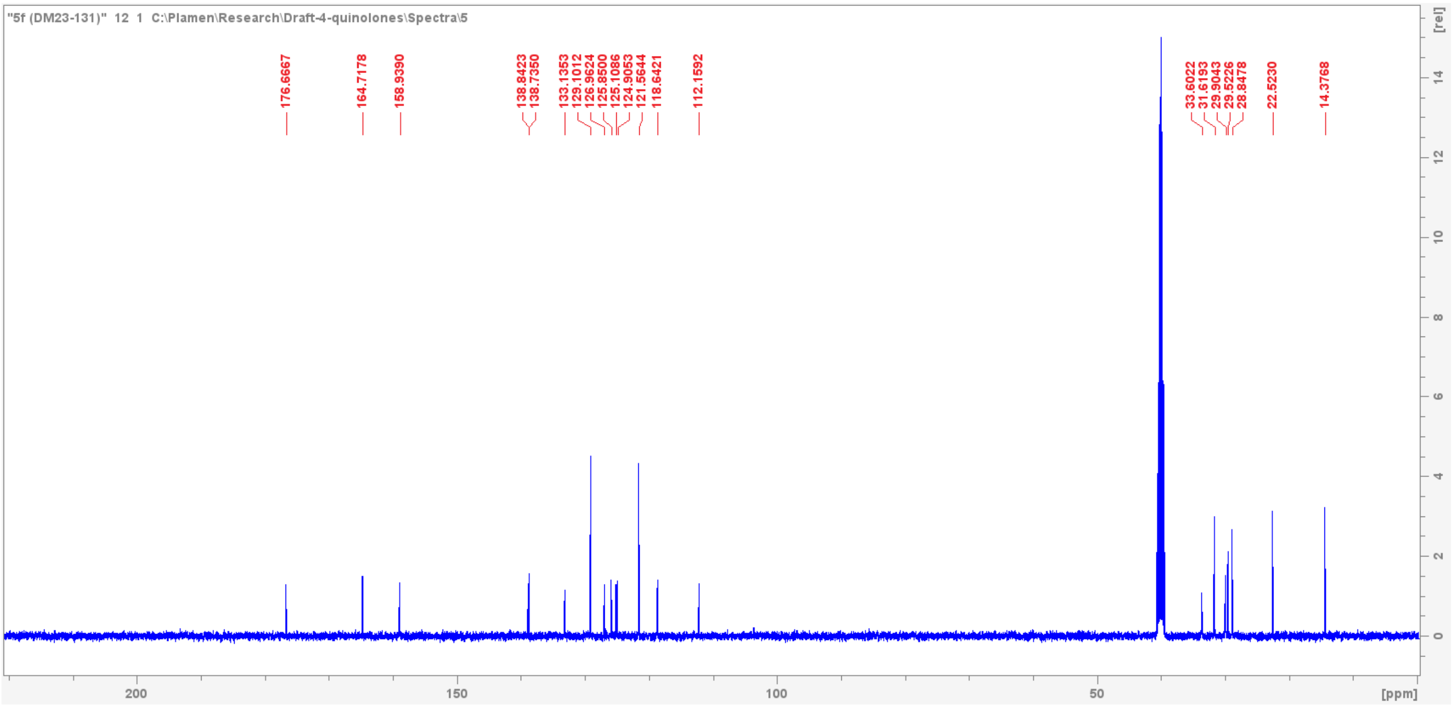

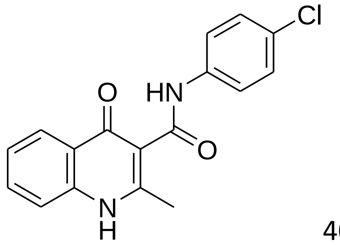

5g

400 MHz, DMSO-d_6

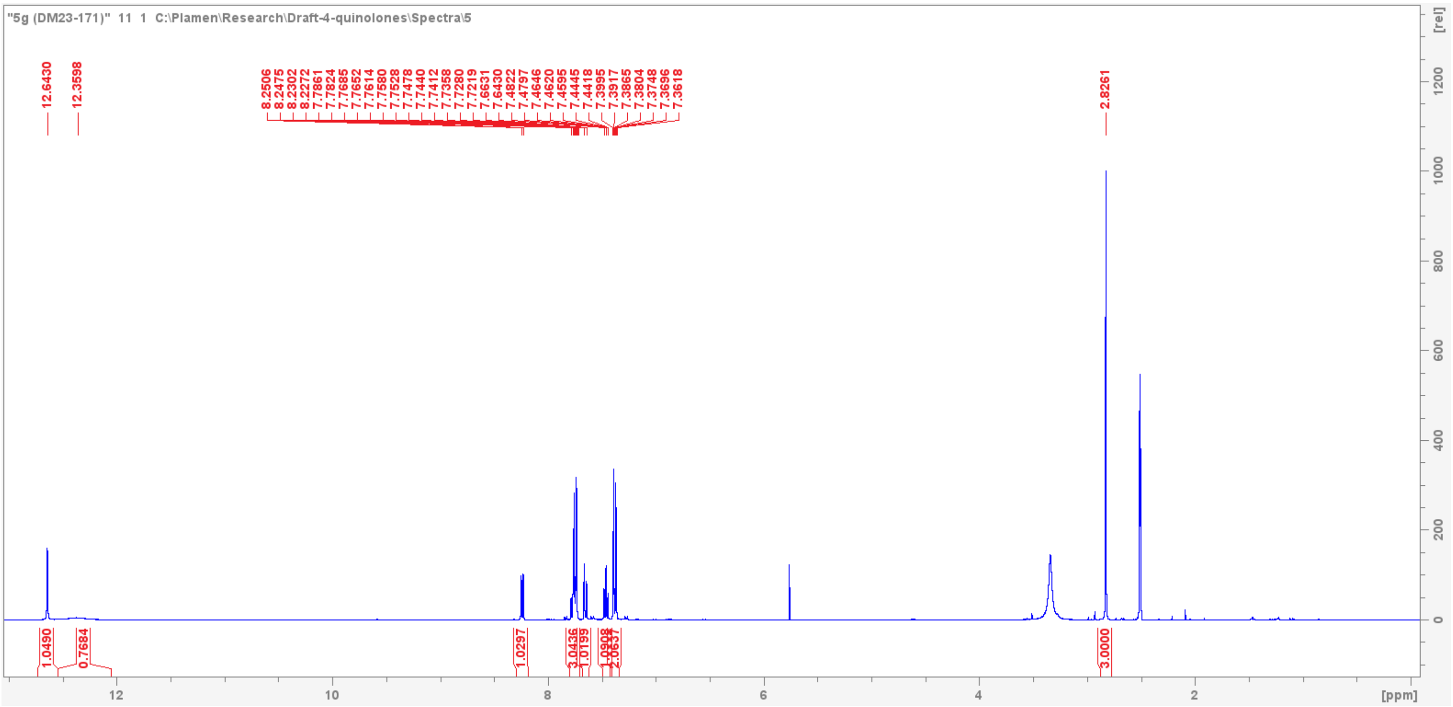

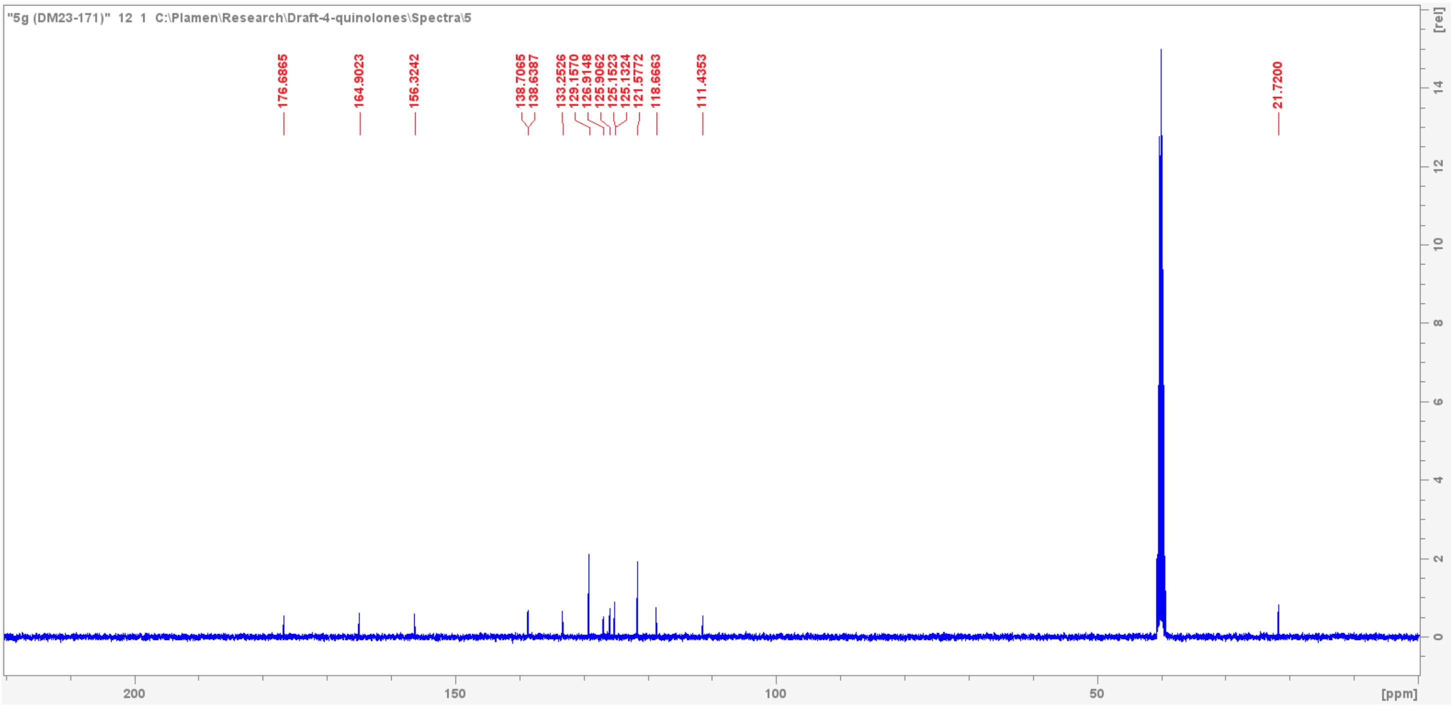

5h 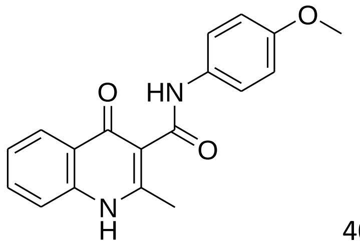 400 MHz, DMSO-d_6

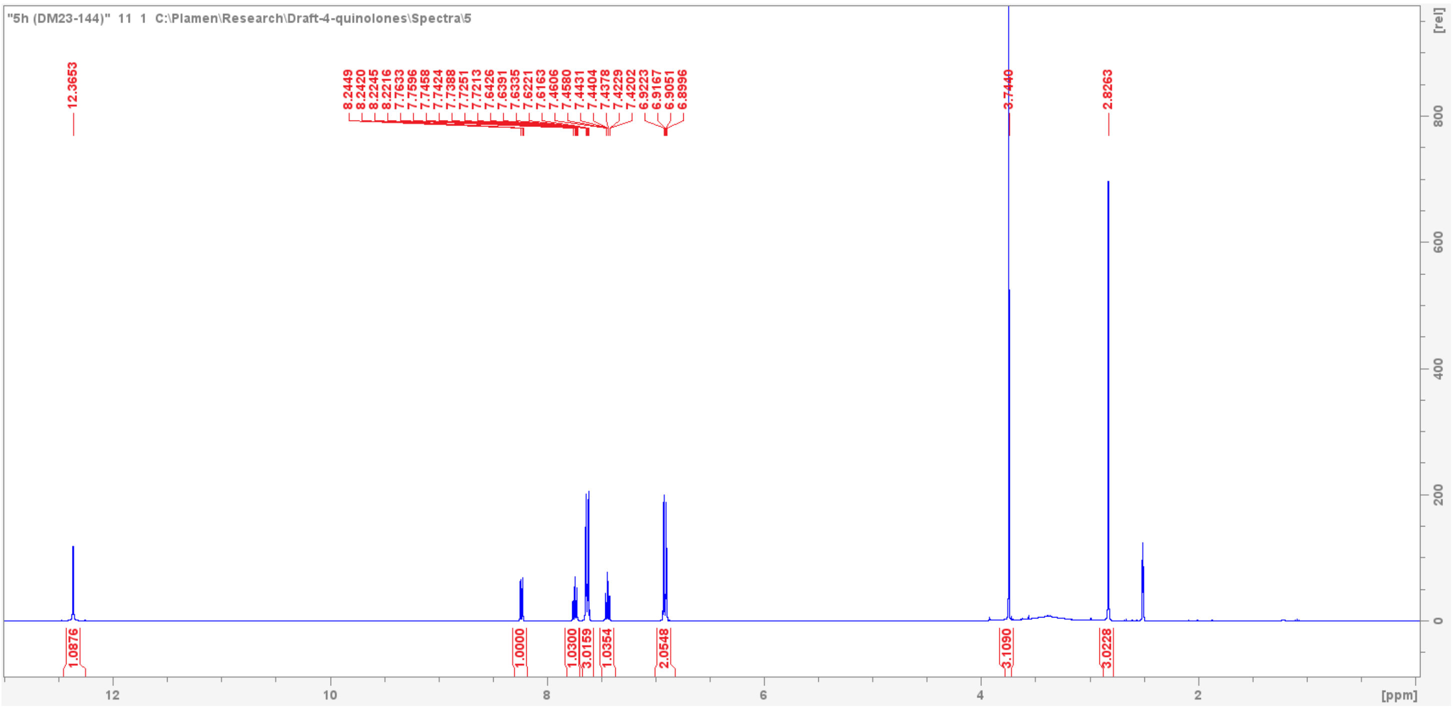

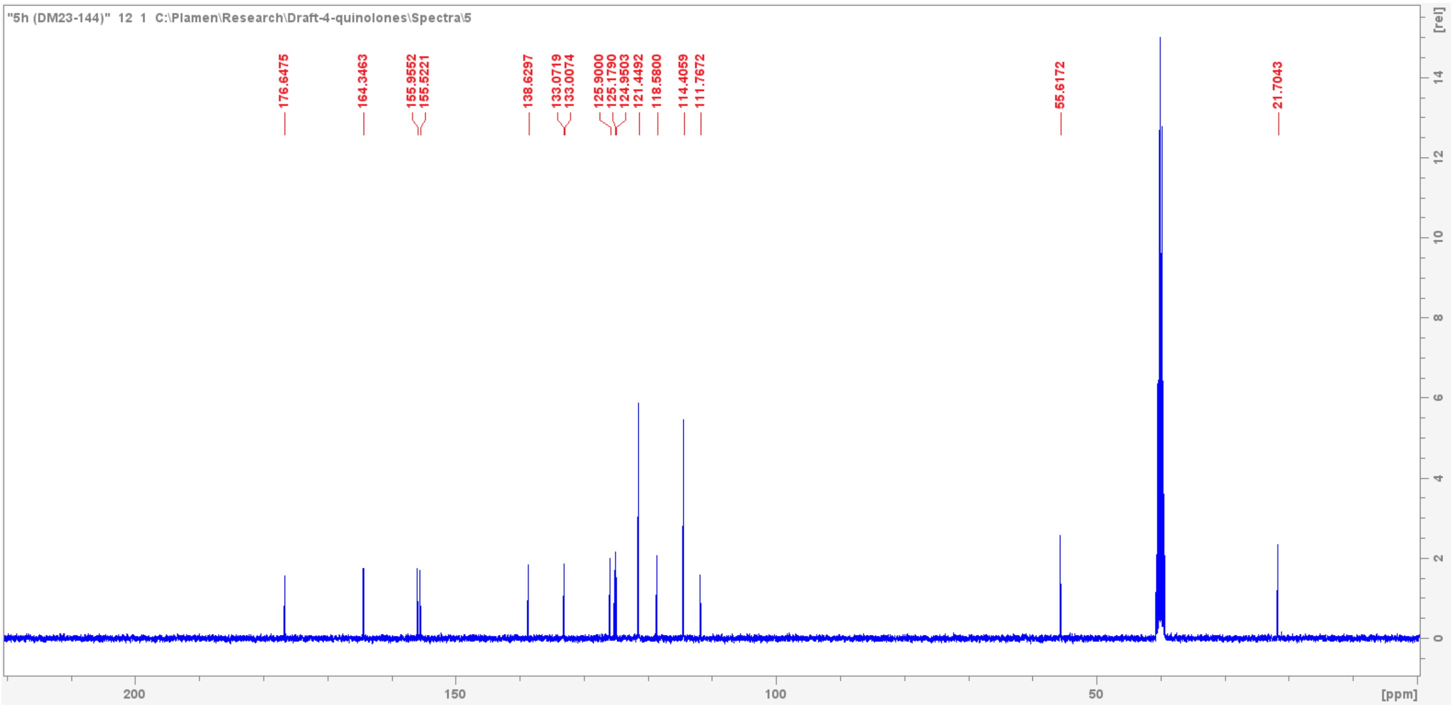

5i 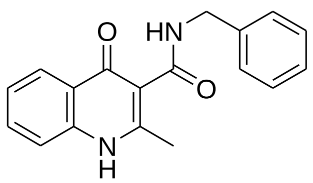 400 MHz, DMSO-d_6

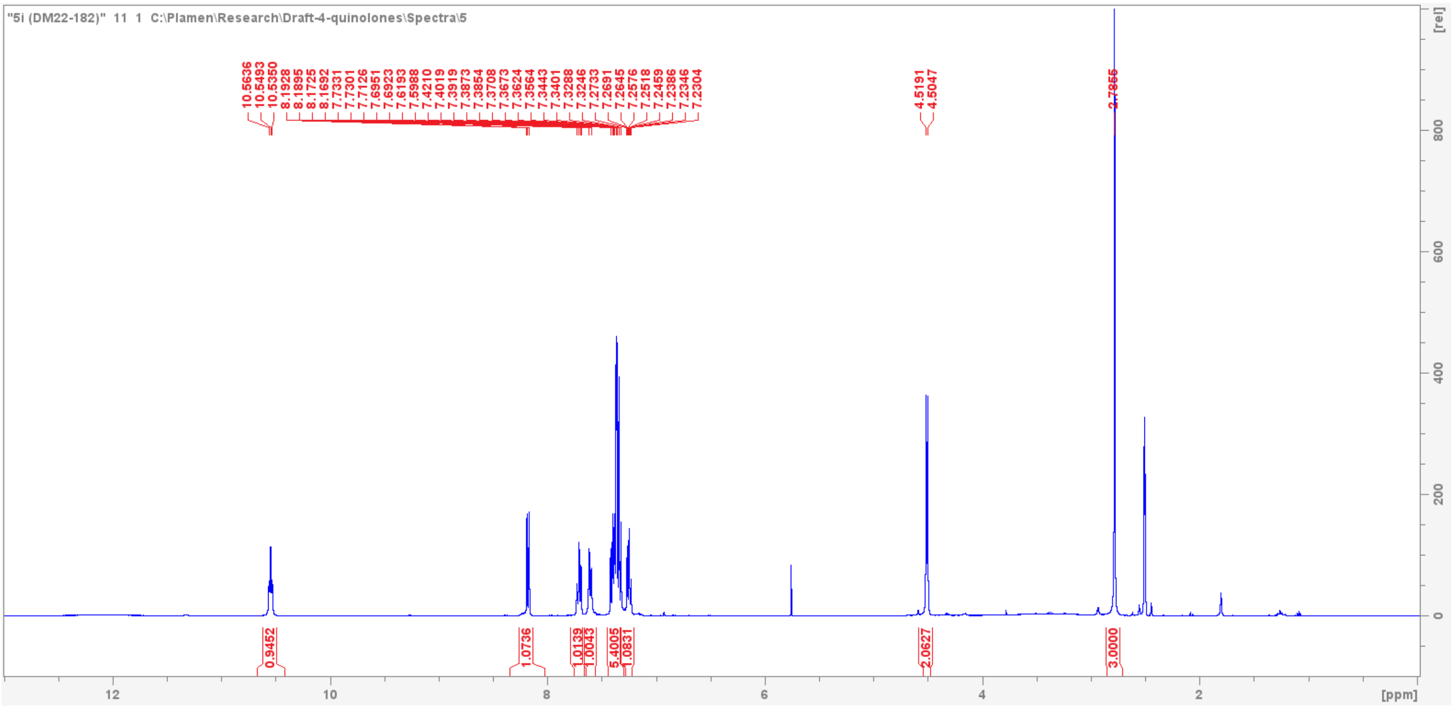

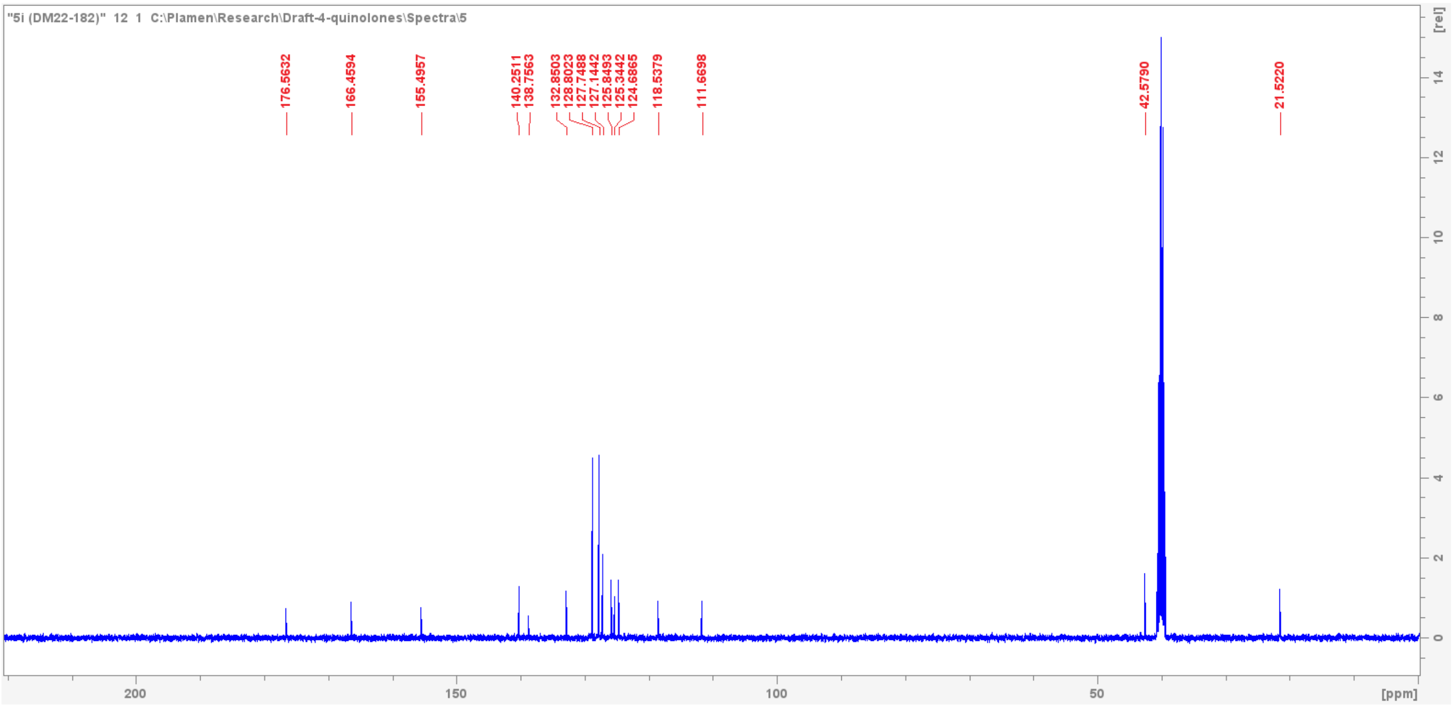

8a 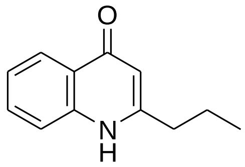

400 MHz, DMSO-d_6

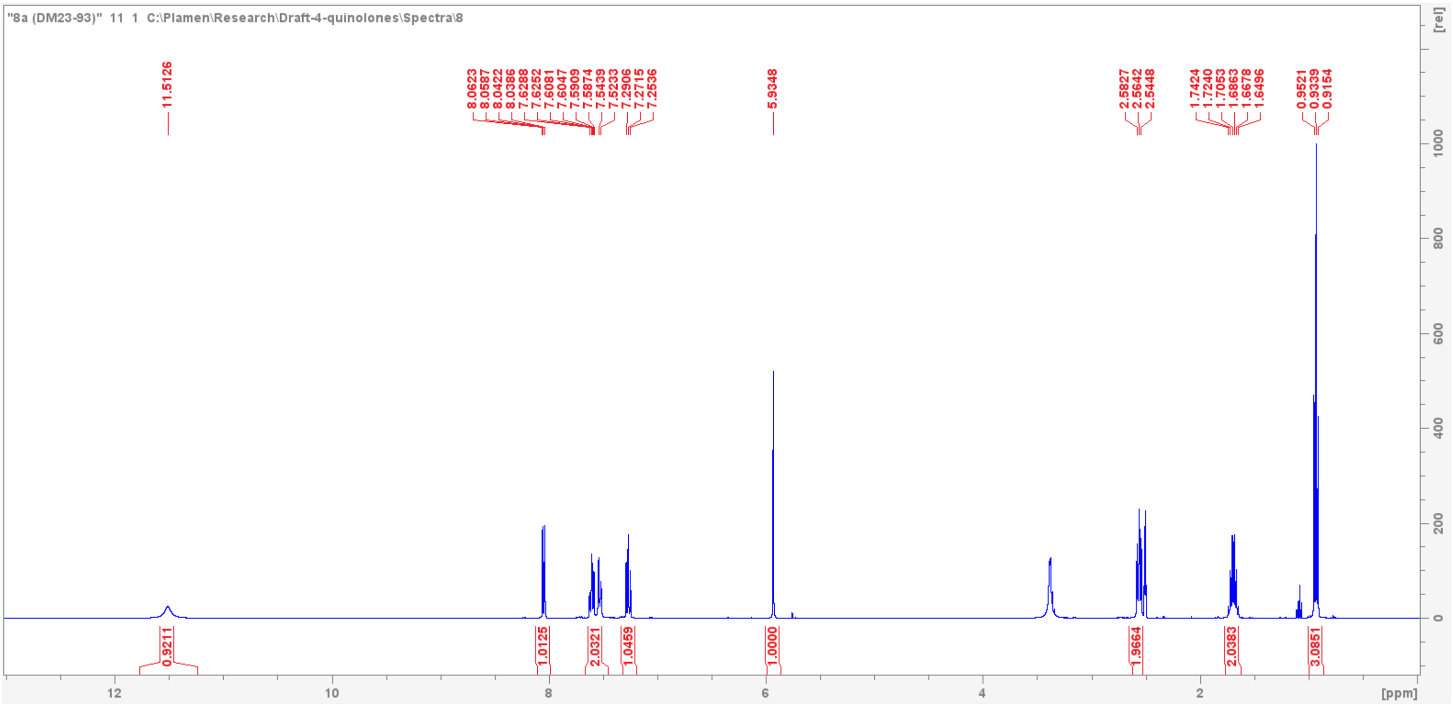

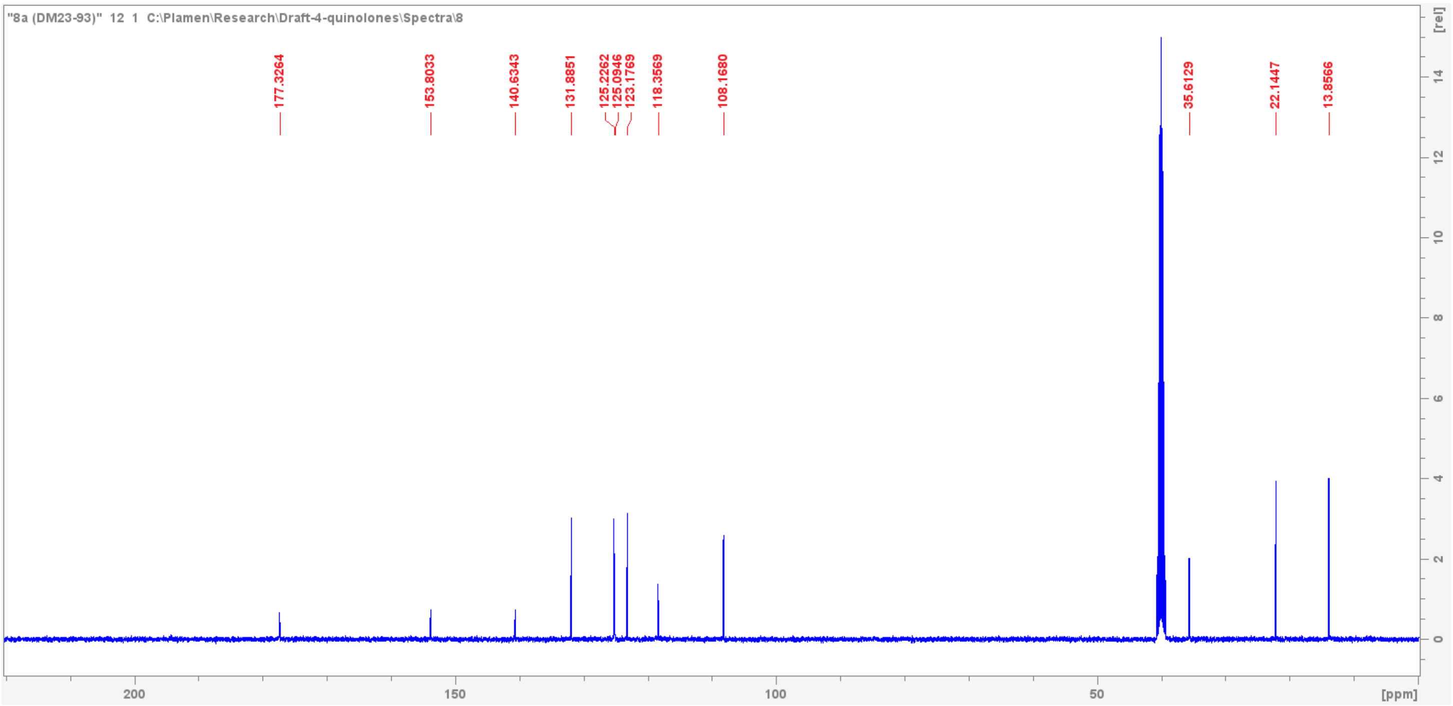

8b 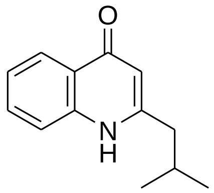

400 MHz, DMSO-d_6

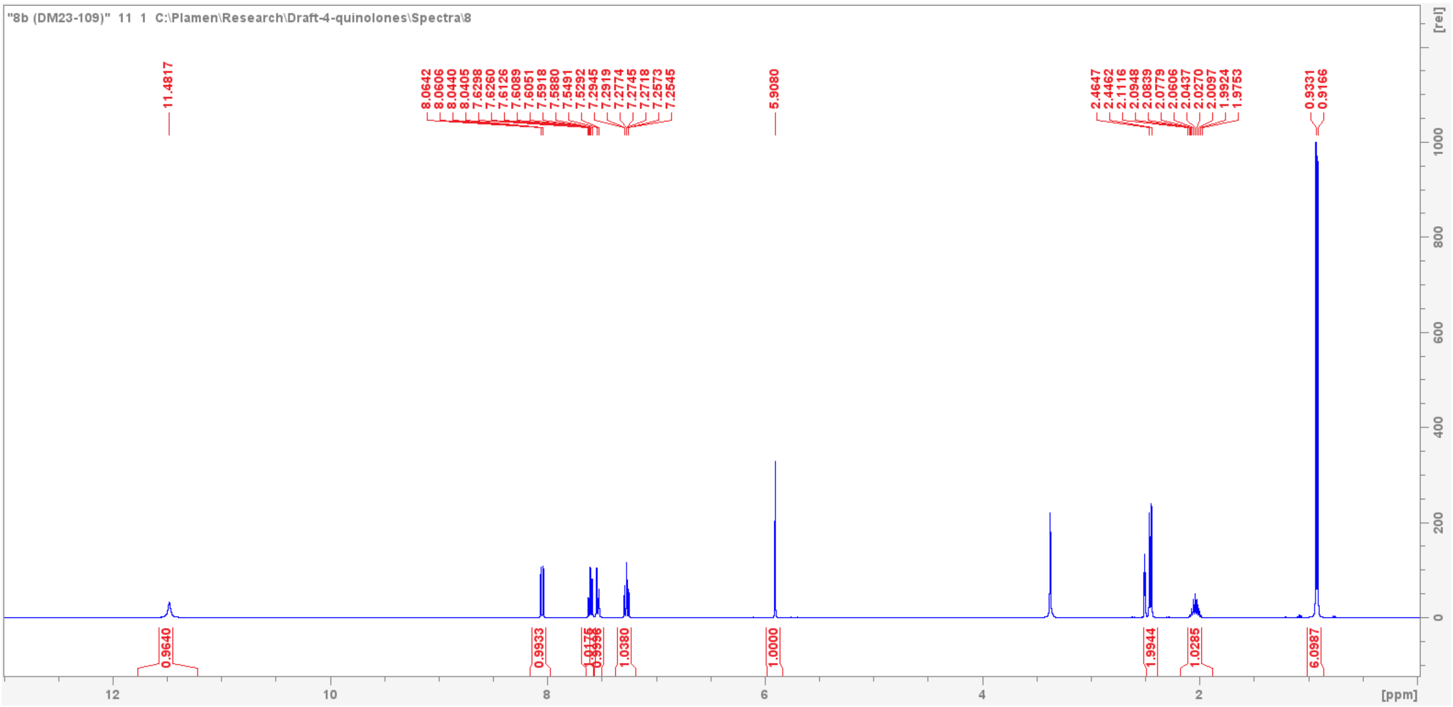

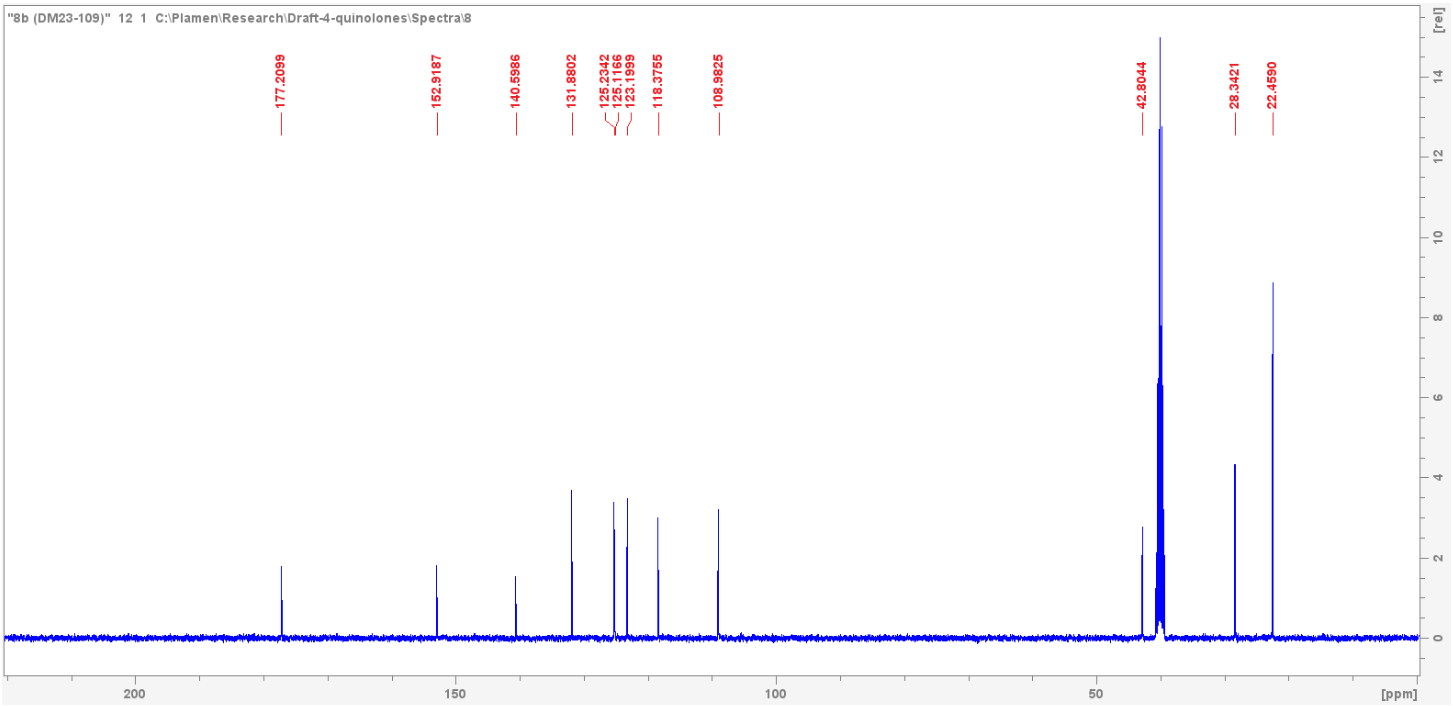

8c 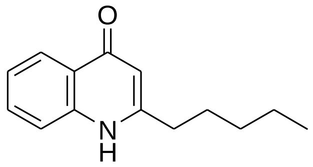 400 MHz, DMSO-d_6

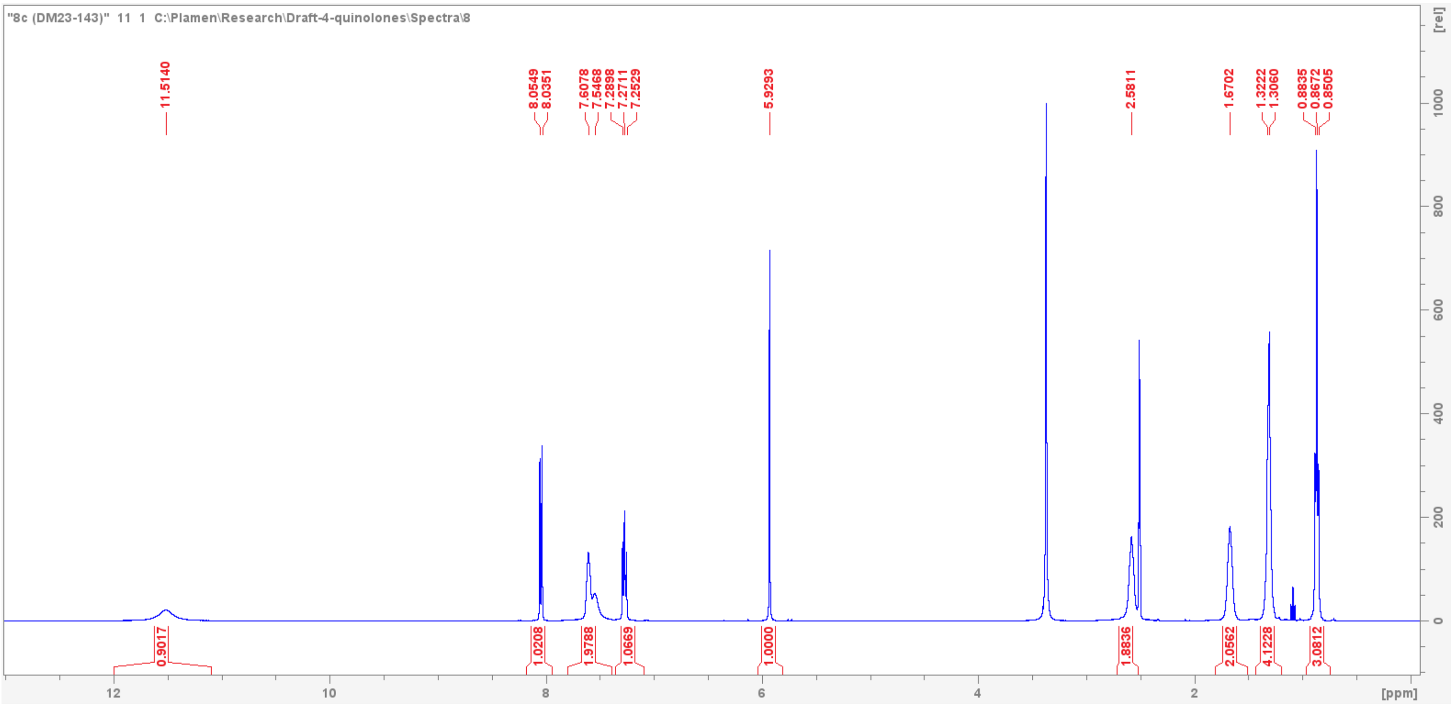

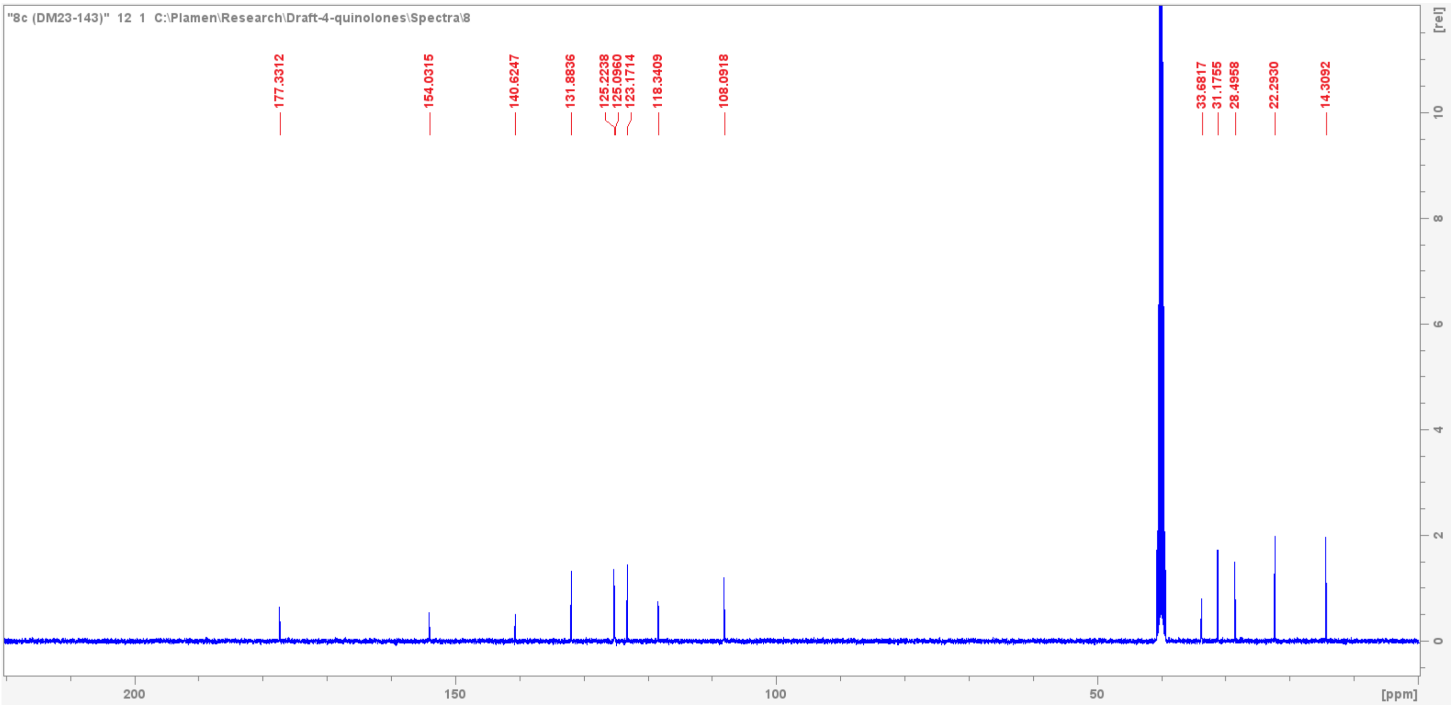

8d 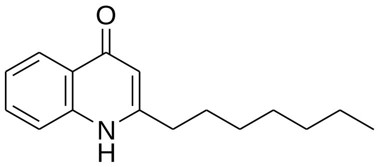

400 MHz, DMSO-d_6

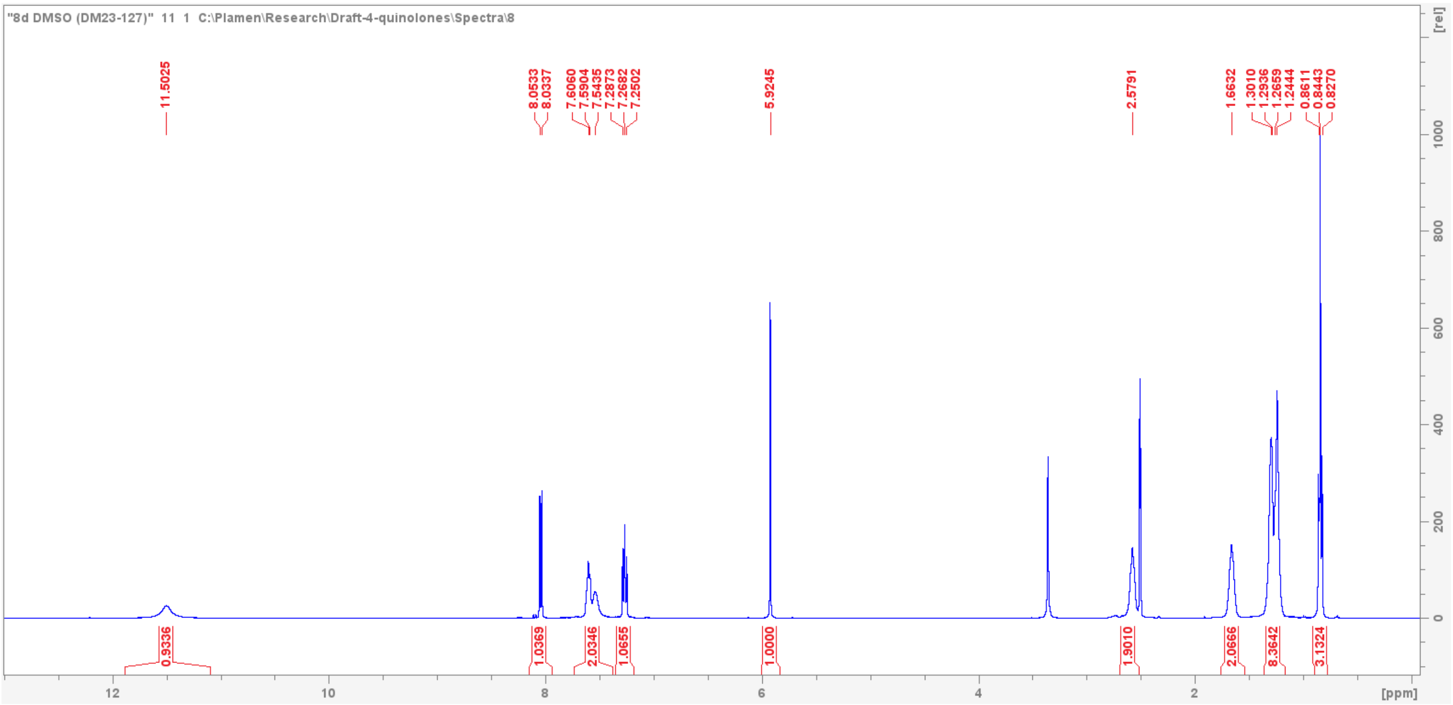

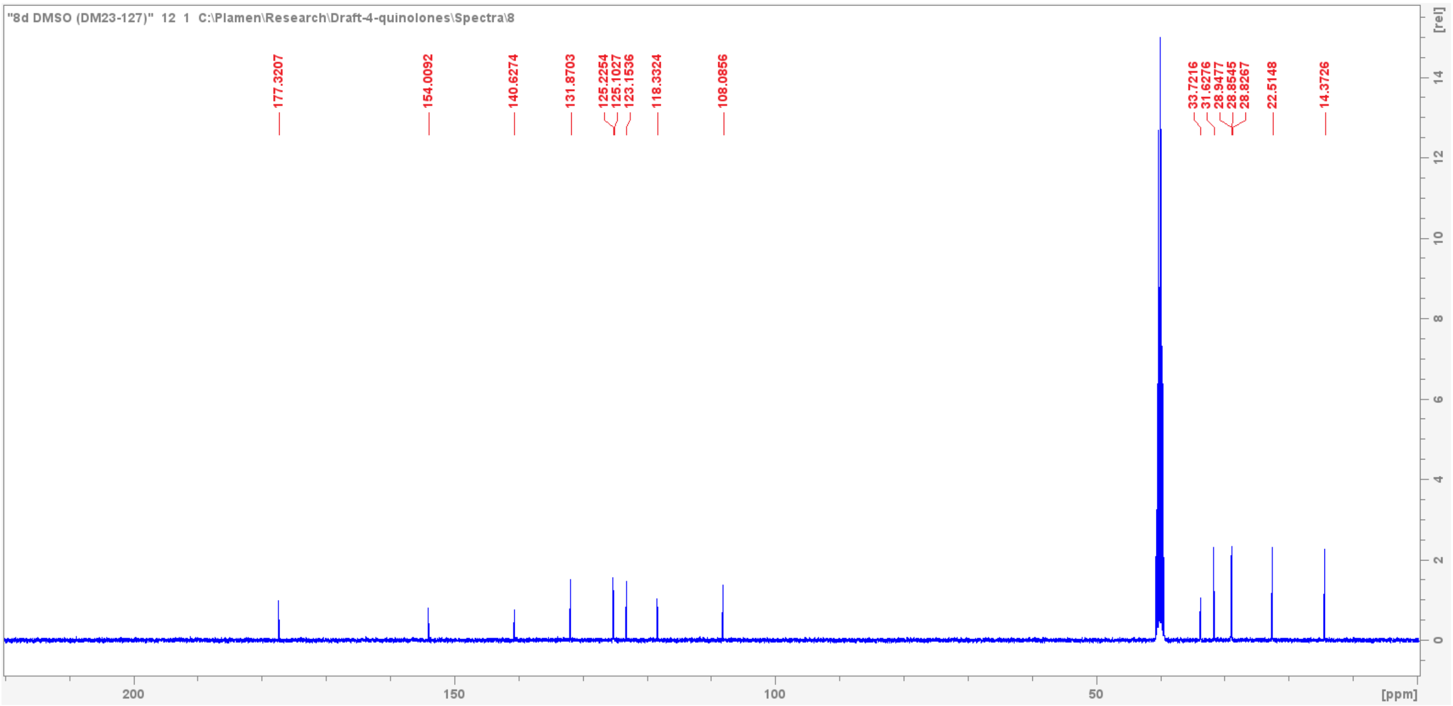

Supplement: File 2 — Processed NMR spectra. [file Beilstein_J_Org_Chem-19-1804-s002.pdf]
